# Tryptophan metabolism in colorectal cancer: From mechanistic insights to novel therapeutic strategies

**DOI:** 10.1002/ctm2.70831

**Published:** 2026-09-14

**Authors:** Ran Wang, Wenke Shen, Ping Yang, Dongxue Yang, Yuepeng Cao, Yuping Zhou

**Affiliations:** ^1^ Department of Gastroenterology The First Affiliated Hospital of Ningbo University Ningbo Zhejiang Province China; ^2^ Health Science Center Ningbo University Ningbo Zhejiang Province China; ^3^ Department of Geriatric Medicine The First Affiliated Hospital of Ningbo University Ningbo Zhejiang Province China; ^4^ Institute of Digestive Disease of Ningbo University Ningbo Zhejiang Province China; ^5^ Laboratory of Translational Medicine Research on Gastroenterology and Hepatology Ningbo Key Laboratory Ningbo Zhejiang Province China; ^6^ Department of Colorectal and Anal Surgery The First Affiliated Hospital of Ningbo University Ningbo Zhejiang Province China; ^7^ Department of Traditional Chinese Medicine The First Affiliated Hospital of Ningbo University Ningbo Zhejiang Province China

**Keywords:** colorectal cancer, diagnostic biomarkers, gut microbiota, indole pathway, kynurenine pathway, prognostic evaluation, serotonin pathway, therapeutic targets, tryptophan metabolism

## Abstract

**Background:**

Tryptophan metabolism drives colorectal cancer (CRC) pathogenesis through three interconnected cascades: the kynurenine pathway, the serotonin pathway, and the gut‐microbiota‐dominated indole pathway. These pathways exert context‐dependent dual activities determined by disease stage, metabolite concentration, cell type and tumor microenvironment. Substrate competition and metabolic flux redistribution occur across the three branches, while extra‐intestinal tryptophan metabolites mediate multi‐organ crosstalk. Despite substantial preclinical findings, clinical translation is hindered by imperfect predictive biomarkers, therapeutic bypass resistance and insufficient causal evidence for the microbiota‐metabolite‐host axis.

**Main body:**

This review summarizes biotransformation features and substrate‐competing networks of tryptophan metabolism. The kynurenine pathway, responsible for approximately 95% of systemic tryptophan catabolism, exerts stage‐dependent dual roles. Its plasma‐ and feces‐derived metabolites and rate‐limiting enzyme indoleamine 2,3‐dioxygenase 1 show diagnostic and prognostic potential, yet monotherapies targeting this enzyme yield poor outcomes due to interleukin‐4‐induced gene 1‐mediated bypass resistance. The serotonin pathway accounts for only 1%‐2% of tryptophan flux; it is predominantly tumor‐promoting and protective solely in early‐phase colitis‐associated tumorigenesis. Selective serotonin reuptake inhibitors hold promise for chemoprevention and immunotherapy sensitization. The microbiota‐governed indole pathway generates diverse compounds: indole‐3‐lactic acid, indole‐3‐acetic acid and indole‐3‐propionic acid boost programmed cell death protein 1 (PD‐1)‐based therapeutic efficacy; indole‐3‐acrylic acid and 3‐methylindole facilitate tumor progression; indole‐3‐acetaldehyde displays concentration‐dependent bidirectional effects. We also discuss natural‐product‐based and microbiota‐targeted interventions, as well as key experimental bottlenecks.

**Conclusion:**

Preclinical metabolite‐guided personalized therapy for CRC requires multi‐omics, stable‐isotope tracing and organoid‐based large‐scale prospective cohorts. Standardized patient stratification and clarification of causal metabolic networks are urgently needed.

## Highlights

1. The kynurenine, serotonin, and indole pathways all modulate CRC progression.
2. Kynurenine pathway molecules serve as diagnostic and prognostic biomarkers and therapeutic targets.
3. The serotonin pathway exerts bidirectional effects dependent on cell type and stage in CRC.
4. Indole metabolites from gut microbiota demonstrate heterogeneous functions and antitumor translational potential.

## Graphical abstract

Tryptophan metabolism modulates CRC via three microbiota‐regulated pathways with distinct dual‐mode effects. The kynurenine pathway provides biomarkers, serotonin pathway supports SSRI‐combined immunotherapy, indole pathway yields immunostimulatory metabolites, collectively offering translational prospects.

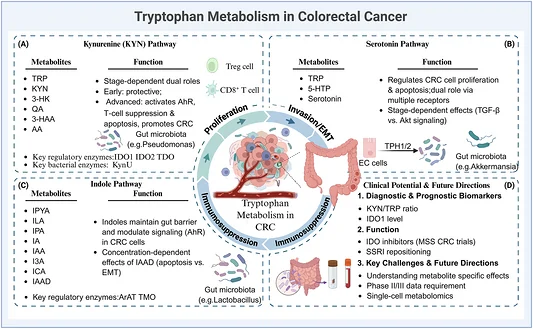

## 1 INTRODUCTION

Colorectal cancer (CRC) remains among the most prevalent malignancies worldwide. Epidemiological data indicate that CRC ranks third in incidence and second in mortality among all cancer types globally.^1^ According to GLOBOCAN 2020 statistics, current trends suggest that the global burden of CRC will increase to 3.2 million new cases and 1.6 million deaths by 2040.^2^ Despite continuous advancements in diagnostic and therapeutic techniques, the mainstay of clinical management for the majority of patients with advanced CRC remains chemotherapy, targeted therapy and immunotherapy. These modalities often face challenges such as resistance and adverse effects in improving long‐term prognosis, largely because of a lack of tumour specificity or the inhibitory effects of tumour microenvironment (TME) immune evasion mechanisms.

On the basis of microsatellite stability, CRC is classified into two major subtypes, mismatch repair‐deficient (dMMR) or microsatellite instability‐high (MSI‐H), which accounts for 15% of cases, and microsatellite stable (MSS) or microsatellite instability‐low (MSI‐L), which accounts for the remaining 85% of cases. Among these, dMMR/MSI‐H tumours exhibit a high tumour mutation burden (TMB) that favours immune cell infiltration, thus increasing the likelihood that patients will derive clinical benefit from immunotherapy.^3^ In recent years, extensive attention has focused on the gut microbiota and its derivatives as novel targets for CRC diagnosis, therapy and reversal of treatment resistance, leading to significant research breakthroughs.

The gut microbiota plays a pivotal role in maintaining host physiological health. By converting host‐ingested nutrients into diverse metabolites, it sustains intestinal homeostasis through mechanisms such as modulating intestinal barrier function and regulating specific signalling pathways.^4^, ^5^ Driven by advancements in bacterial sequencing and isolation–culture techniques, research into the links between the gut microbiota and human diseases has accelerated, gradually elucidating its dual role in maintaining homeostasis and driving pathogenesis. Microbiota‐derived metabolites, which serve as critical mediators in the cross‐talk between bacteria and the host, have emerged as a focal point in mechanistic investigations of various diseases. To date, however, only a limited number of these metabolites have been extensively characterized, such as short‐chain fatty acids (SCFAs), secondary bile acids (SBAs), and trimethylamine N‐oxide (TMAO).^6^, ^7^, ^8^ More recently, metabolic pathways involving aromatic amino acids, particularly tryptophan, have garnered significant interest, offering novel insights into the complex regulatory networks through which the microbiota influences host homeostasis and disease states.

As an essential aromatic amino acid, tryptophan (TRP) lacks an endogenous biosynthetic pathway and must be acquired through dietary intake. Consequently, the host relies on intestinal absorption and microbial metabolism to maintain systemic tryptophan homeostasis. Beyond its role in protein synthesis, tryptophan serves as a precursor for various bioactive metabolites, including serotonin, melatonin and vitamin B3.^9^, ^10^, ^11^, ^12^ Tryptophan can be catabolized into diverse metabolites by the gut microbiota and various key enzymes, playing pivotal roles in the pathogenesis, progression, and therapeutic intervention of numerous disorders, including digestive and neurological diseases.^13^, ^14^ Thus, investigating tryptophan and its downstream metabolites not only can elucidate the underlying mechanisms of disease but also provide a theoretical foundation for the development of novel therapeutic strategies targeting metabolic enzymes or receptors. In this review, we summarize the functional roles and clinical implications of tryptophan metabolism in the context of colorectal cancer.

## 2 BIOTRANSFORMATION PROCESSES OF TRYPTOPHAN METABOLISM

In vivo, tryptophan is primarily catabolized via three major metabolic routes: the kynurenine (KYN) pathway, the serotonin pathway and the microbial indole metabolic pathway.^15^

### 2.1 Biotransformation Processes of the Kynurenine Pathway

The metabolic process of the kynurenine pathway (KP) involves multiple enzymatic reactions, in which the expression or activity of key enzymes dictates metabolite production and subsequently modulates CRC progression. Initially, tryptophan is converted into N‐formylkynurenine (NFK) by indoleamine 2,3‐dioxygenase 1/2 (IDO1/IDO2) and tryptophan 2,3‐dioxygenase (TDO). NFK is subsequently hydrolysed into kynurenine via the catalytic action of arylformamidase (AFMID).^16^ Kynurenine serves as a substrate for further transformation into diverse downstream metabolites. Specifically, kynurenine‐3‐monooxygenase (KMO) catalyses the conversion of KYN into 3‐hydroxykynurenine (3‐HK). Kynureninase (KYNU) subsequently facilitates the production of multiple metabolites, including 3‐hydroxyanthranilic acid (3‐HAA), quinolinic acid (QA), and xanthurenic acid (XA). Alternatively, kynurenine aminotransferases I‐IV (KAT I‐IV) catalyse the conversion of kynurenine into kynurenic acid (KYNA). Moreover, KYNU can convert kynurenine into anthranilic acid (AA).^17^

IDO1/IDO2 serve as the primary rate‐limiting enzymes initiating the kynurenine metabolic pathway. Under physiological conditions, IDO expression is low or negligible across most human tissues. Its expression is predominantly induced by proinflammatory cytokines, including interleukin‐6 (IL‐6) and tumour necrosis factor‐α (TNF‐α).^18^, ^19^ In addition to IDO, tryptophan 2,3‐dioxygenase (TDO), which is specifically expressed in the liver, catalyses the conversion of TRP into KYN, a metabolite with potent immunosuppressive activity. This process contributes to the maintenance of systemic immune homeostasis.

The gut microbiota can modulate the activity of these rate‐limiting enzymes, thus promoting or suppressing. For instance, studies have demonstrated that the intestinal commensal bacterium *Achromobacter pulmonis* can increase IDO1 activity. This enhancement facilitates the conversion of TRP into KYN, subsequently modulating the activation of the aryl hydrocarbon receptor (AhR).^20^

### 2.2 Biotransformation Processes of the Serotonin Pathway

As the precursor for serotonin synthesis, TRP is primarily converted into serotonin within the gut via two distinct pathways: (1) approximately 95% of TRP is processed in enterochromaffin cells (ECs) through catalysis by tryptophan hydroxylase 1 (TPH1) to generate 5‐hydroxytryptophan (5‐HTP), which subsequently undergoes decarboxylation by aromatic amino acid decarboxylase (AADC) to form serotonin, representing the predominant source of intestinal serotonin; (2) a minor proportion of serotonin is synthesized in the enteric nervous system (ENS) under the mediation of tryptophan hydroxylase 2 (TPH2).^21^ Emerging evidence indicates that the gut microbiota and its derived metabolites, along with various nutrients and hormones, can modulate serotonin secretion by EC cells, thus influencing systemic serotonin levels.^22^, ^23^

The gut microbiota can modulate the synthesis and utilization of host serotonin via a diverse array of molecular mechanisms.^24^ For instance, it has been demonstrated that *Escherichia coli* expresses tryptophan synthase, thus supplying the essential substrate for serotonin production and facilitating host serotonin synthesis.^25^ Furthermore, following intervention with *Akkermansia muciniphila*, murine colonic tissues exhibit upregulated TPH1 expression and downregulated serotonin reuptake transporter (*SERT*) expression, suggesting that *Akkermansia muciniphila* can potentiate serotonin biosynthesis and increase its extracellular availability.^26^

### 2.3 Biotransformation Processes of the Microbial Indole Pathway

The indole metabolic pathway comprises a diverse array of enzymatic reactions. Initially, tryptophan is converted into indole‐3‐pyruvic acid (IPYA) via the catalytic action of aromatic amino acid transaminase (ArAT).^8^ IPYA undergoes further transformation mediated by the gut microbiota through the following routes: (1) IPYA can be reduced to indole‐3‐lactic acid (ILA), which is subsequently metabolized into indole acrylic acid (IA) and indole‐3‐propionic acid (IPA). (2) IPYA can also be converted into indole‐3‐acetaldehyde (IAAD), a key intermediate that yields indole‐3‐acetic acid (IAA), indole‐3‐aldehyde (I3A), and indole‐3‐carboxylic acid (ICA). Furthermore, TRP can be catalysed by tryptophan 2‐monooxygenase (TMO) to produce indole‐3‐acetamide (IAM), which is subsequently transformed into IAA.^27^

### 2.4 Multi‐organ regulatory networks of tryptophan metabolism

Notably, while most of the current research focuses on the localized regulation of tryptophan metabolism within the gut, Tang et al. systematically integrated a multi‐organ tryptophan metabolic network, establishing a crucial theoretical framework for understanding host–microbiota cross‐talk. Certain tryptophan metabolites may facilitate cross‐organ signal transduction and distal regulation via the systemic circulation, operating through multi‐organ regulatory axes such as the gut–brain, gut–liver, gut–lung and gut–kidney axes.^28^

**FIGURE 1:**
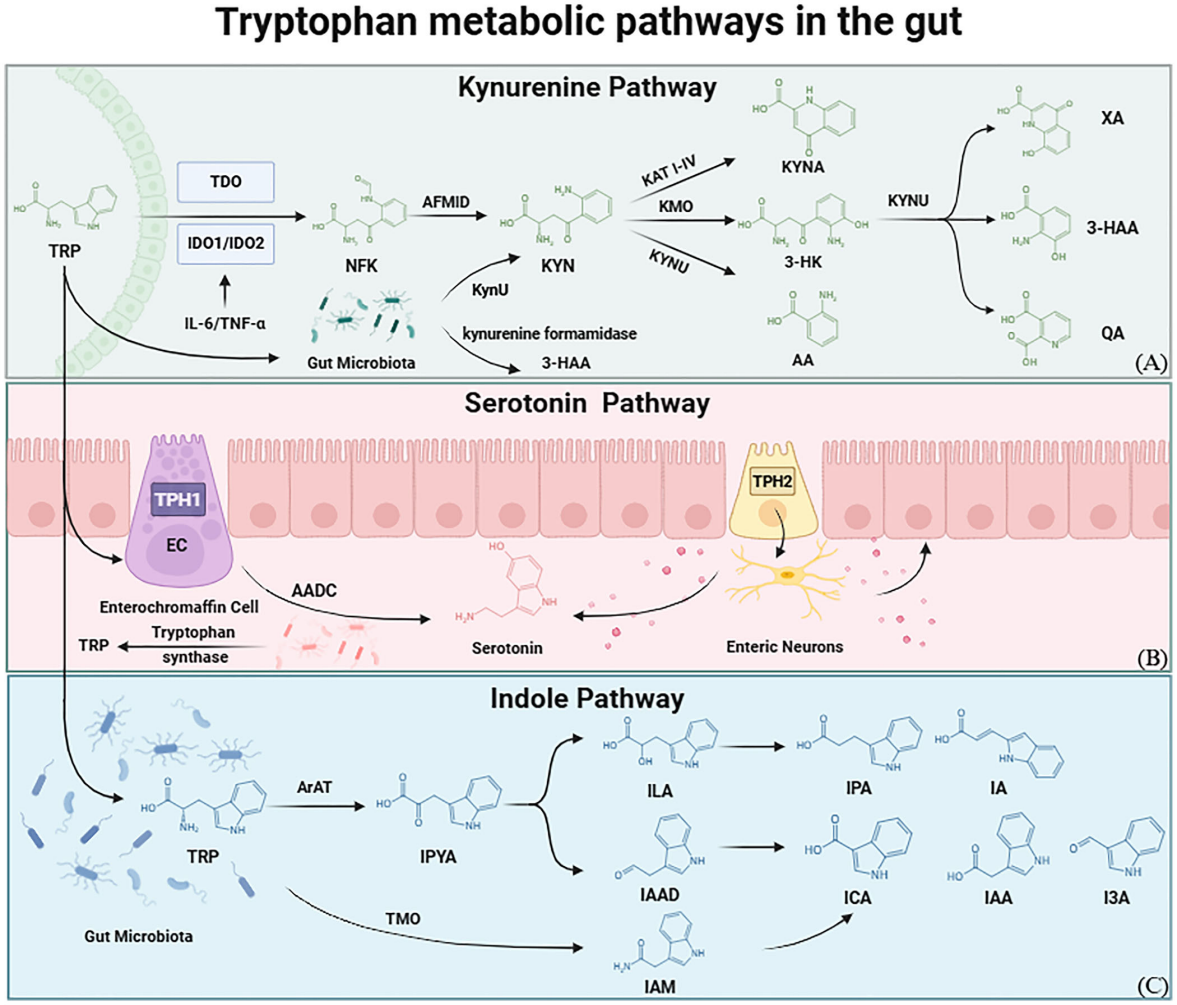
Tryptophan metabolic pathways in the gut. Tryptophan (TRP) is primarily metabolized via three major pathways in the gut, and gut microbiota participate in and modulate all three pathways: (A) Kynurenine (KYN) pathway: Catalysed by host enzymes tryptophan 2,3‐dioxygenase (TDO) and indoleamine 2,3‐dioxygenase 1/2 (IDO1/IDO2), TRP is converted into N‐formylkynurenine (NFK) and subsequently KYN. The expression of IDO1 and IDO2 can be induced by proinflammatory cytokines such as IL‐6 and TNF‐α. KYN is further metabolized into various downstream derivatives including 3‐hydroxykynurenine (3‐HK), kynurenic acid (KYNA), and quinolinic acid (QA) through enzymes such as kynurenine aminotransferase I—IV (KAT I‐IV), kynurenine 3‐monooxygenase (KMO), and kynureninase (KYNU). Gut microbiota also contribute to this pathway via specific metabolic enzymes including kynureninase (KynU) and kynurenine formamidase. (B) Serotonin pathway: Gut microbiota generate tryptophan via tryptophan synthase to provide substrate for serotonin production. In enterochromaffin cells (ECs), TRP is primarily converted into serotonin by tryptophan hydroxylase 1 (TPH1) and aromatic amino acid decarboxylase (AADC). A minor fraction of serotonin is also synthesized in enteric neurons via tryptophan hydroxylase 2 (TPH2). (C) Indole pathway: Driven entirely by gut microbiota, TRP is metabolized into diverse indole derivatives such as indole‐3‐propionic acid (IPA) and indole‐3‐acetic acid (IAA), which mediate crosstalk between the host and microbiota.

Building upon the aforementioned evidence of multi‐organ cross‐talk, we postulate that extra‐intestinal tryptophan metabolites might participate in the initiation and progression of colorectal cancer. On the one hand, as the primary organ expressing TDO, the liver synthesizes kynurenine, which can potentially reach the colon via portal circulation, thus modulating the tumour microenvironment. On the other hand, IDO, a critical enzyme broadly distributed across diverse tissues, mediates immunosuppressive effects that may extend beyond the local tumour milieu, potentially attenuating systemic anti‐tumour immune responses via the bloodstream. Previous studies have shown that IDO drives the expansion of regulatory T cells (Tregs) and myeloid‐derived suppressor cells (MDSCs) to foster an immunosuppressive tumour microenvironment, thus mediating immunosenescence.^29^ Should these effects be exerted on the colon via the circulatory system, they could further facilitate the immune evasion of colorectal cancer. The metabolic conversion processes of tryptophan in the gut, together with key enzymes and downstream metabolites, are illustrated in Figure 1.

Furthermore, I3A derived from the maternal gut microbiota can be transferred to neonates via the placenta or breast milk, thus activating AhR signalling in their innate lymphoid cells and contributing to the postnatal development of the intestinal immune system.^30^ These findings directly corroborate the capacity of tryptophan metabolites to facilitate long‐distance, cross‐organ signal transduction via the systemic circulation; concurrently, these findings suggest that tryptophan metabolites generated in extra‐intestinal organs could similarly reach the colon via the bloodstream to participate in the modulation of immune homeostasis in colorectal cancer.

### 2.5 Substrate competition and flux redistribution among TRP metabolic pathways

As an essential amino acid incapable of endogenous synthesis, TRP represents a finite substrate within the colorectal microenvironment; consequently, the kynurenine, serotonin, and indole metabolic pathways do not operate as isolated parallel systems but rather engage in continuous cross‐talk characterized by substrate competition and dynamic metabolic flux redistribution. Thus, rather than functioning strictly independently, these three cascades exist in a constant state of dynamic substrate competition and flux reallocation. Alterations in the activity of rate‐limiting enzymes within a specific pathway subsequently shift the distribution of free TRP, thus modulating metabolic flux through alternative branches; however, this compensatory cross‐pathway effect is governed by multiple variables, including tissue compartmentalization, specific enzyme kinetics and the composition of the gut microbiota.

Upon aberrant activation of IDO1/TDO, a substantial proportion of free TRP is shunted toward the KP, directly depleting the substrate reservoir available for serotonin synthesis by enterochromaffin cells and indole derivative production by the gut microbiota. Conversely, excessive consumption of luminal TRP by the gut microbiota for indole synthesis, or sustained high expression of TPH1 driving serotonin production, can sequester the substrate and subsequently dampen the KP. Current evidence indicates that high‐fat diet‐induced obesity can upregulate intestinal IDO activity, thus skewing TRP metabolism away from indole and IL‐22‐associated pathways toward the KP.^31^ Pro‐inflammatory cytokines, such as IL‐1β and TNF‐α, can trigger IDO activation and subsequent systemic free TRP depletion, which in turn diminishes serotonin biosynthesis and bioavailability; these findings suggest that inflammation‐associated serotonin deficits stem from TRP substrate shunting rather than absolute amino acid deficiency.^32^ Similarly, IDO hyperactivation relentlessly consumes TRP, further constraining the generation of serotonin and microbiota‐derived indole derivatives.^33^ In contrast, microbiota‐derived indole derivatives consume luminal TRP substrates while concurrently modulating host IDO1 and AhR signalling to suppress KP flux.^34^

The metabolic flux of TRP across the three pathways is jointly regulated by spatial segregation, rate‐limiting enzymes, the microenvironment and the gut microbiota. Specifically, the metabolic shunting of TRP is governed by three primary factors. (1) Spatial segregation: The kynurenine and serotonin pathways predominantly occur in diverse host somatic cells, whereas the indole pathway is restricted to the gut microbiota within the intestinal lumen. Furthermore, distinct capacities for TRP transport and uptake among tumour cells, immune cells, intestinal epithelial cells, and the gut microbiota constitute a spatial barrier for substrate partitioning. (2) Differential expression of rate‐limiting enzymes: The expression and activation levels of hepatic TDO, IDO1, intestinal epithelial TPH1, and microbial tryptophan‐metabolizing enzymes directly dictate the consumption rate of TRP. (3) Microenvironment and microbiota: The composition of the gut microbiota, the relative abundance of pro‐tumorigenic versus tumour‐suppressive taxa, and intestinal barrier integrity further modulate the metabolic flux of TRP.

Enterochromaffin cells and brainstem neurons specifically express TRPH1/2, thus promoting the conversion of TRP into serotonin and melatonin. The liver constitutively expresses high levels of TDO, whereas immune cells can upregulate IDO1 upon inflammatory stimulation, cooperatively redirecting TRP toward the KP; upon entry into the intestinal microenvironment, TRP is metabolized by microbial tryptophanases to generate indole derivatives, including ILA and IPA. Additionally, competition for amino acid transporters and prevailing inflammatory levels further modulate TRP substrate competition and metabolic reallocation.^35^

In summary, the three metabolic pathways of tryptophan undergo biotransformation through intricate enzymatic reactions, with the gut microbiota serving as a pivotal regulator in each route. Bioactive molecules derived from these pathways, including downstream kynurenine metabolites, serotonin, and indole derivatives, play fundamental roles in the pathogenesis, progression, diagnosis and therapeutic intervention of CRC. A comprehensive understanding of the molecular mechanisms through which these metabolites exert their effects is essential for elucidating the intricate relationship between tryptophan metabolism and CRC. Furthermore, such insights provide a solid theoretical foundation for the identification of novel diagnostic biomarkers and therapeutic targets.

## 3 THE KP AND COLORECTAL CANCER

The KP represents the major route of tryptophan catabolism, comprising approximately 95% of the total tryptophan flux.^15^ Accumulating evidence in recent years suggests that KP metabolic disturbances are intimately linked to a variety of gastrointestinal disorders. Beyond being involved in CRC pathogenesis, the KP produces downstream metabolites that modulate immune homeostasis and the tumour microenvironment via complex molecular mechanisms, positioning the KP and its metabolites as prominent areas of contemporary research.^36^

As a multifactorial pathological process, CRC progression has been shown by numerous clinical, in vivo, and in vitro studies to be driven by the KP through diverse molecular pathways, including aryl hydrocarbon receptor (AhR) activation and immune balance regulation.^37^, ^38^ In addition, the relationship between kynurenine metabolism and CRC has been widely investigated, spanning critical domains, including pathogenesis, diagnostic biomarkers, prognostic evaluation and the identification of therapeutic targets.

AhR is a ligand‐activated transcription factor that functions as a metabolite sensor and immunomodulator, and is highly expressed in gastrointestinal epithelial tissues.^39^ Evidence indicates that AhR serves as a critical target through which tryptophan metabolites exert their biological effects. Diverse TRP metabolites can bind to AhR to initiate downstream signalling cascades, thus regulating physiological and pathological processes such as metabolism, inflammation and immune responses and playing a vital role in maintaining intestinal barrier integrity and immune homeostasis.^37^, ^40^, ^41^ Primarily, AhR exhibits pronounced ligand bias, characterized by distinct binding modalities and activation potencies among various ligands. For instance, the exogenous high‐affinity ligand dioxin (TCDD) induces sustained AhR activation, facilitating the formation of a stable AhR‐ARNT complex to initiate the transcription of downstream target genes. Conversely, ligands such as 6‐formylindolo[3,2‐b]carbazole (FICZ) and I3C display weaker binding stability with AhR, thus eliciting only transient activation effects. Within the KP, kynurenic acid and xanthurenic acid demonstrate substantially higher AhR activation efficacy than kynurenine does.^42^ These divergent binding modalities may partially explain the diametrically opposed biological functions resulting from AhR activation.^39^ Second, AhR signalling is highly concentration dependent. Insufficient ligand concentrations lead to defective AhR activation, disrupting the intestinal barrier and precipitating inflammation; optimal concentrations induce moderate AhR activation to maintain intestinal immune homeostasis; conversely, chronic high‐dose ligand exposure triggers pathway hyperactivation, inducing mucosal damage and potentially exerting pro‐tumorigenic effects. The majority of endogenous ligands derived from dietary sources or the viable microbiota additionally exhibit biphasic properties, acting as agonists at low concentrations and antagonists at high concentrations.^43^ Third, AhR activation is intrinsically dependent on the cellular context. Even upon stimulation with an identical ligand, the downstream consequences of AhR activation can vary profoundly depending on the specific cell type. In dendritic cells, AhR activation diminishes the expression of MHC class II antigen‐presenting molecules and CD80/CD86 co‐stimulatory molecules while downregulating the expression of the pro‐inflammatory cytokines IL‐6 and TNF to suppress T‐cell responses; notably, the AhR antagonist StemRegenin 1 (SR1) can reverse this immunosuppressive phenotype.^44^ Melanoma‐related studies further corroborate this hypothesis: AhR expression in tumour cells inhibits proliferation and metastasis, whereas AhR activation in stromal cells accelerates tumour progression.^45^ Furthermore, variables such as the cell cycle, redox status and intercellular interactions may also modulate AhR‐mediated signalling cascades.

Collectively, the highly heterogeneous responsiveness of AhR to its ligands reconciles the long‐standing dichotomy regarding its dual tumour‐promoting and tumour‐suppressive roles in cancer biology.^46^ Accumulating evidence has demonstrated that diverse tryptophan metabolites function as endogenous AhR ligands; consequently, variations in ligand identity, local microenvironmental concentration, and target cell type collectively dictate the divergent biological activities of these metabolites in colorectal cancer.

### 3.1 Dual roles of the KP in CRC

The KP exerts dual tumour‐promoting and tumour‐suppressive effects in the context of CRC. During the early stages of inflammation, moderate activation of the KP generates protective metabolites, such as XA, which mediate anti‐inflammatory and barrier‐protective functions; conversely, as CRC advances to the stage of overt tumorigenesis, the sustained hyperactivation of the KP yields an abundance of immunosuppressive metabolites, thus driving pro‐tumorigenic outcomes.

#### 3.1.1 Pro‐tumorigenic effects and underlying mechanisms of the KP

In the context of KP‐mediated immunomodulation, regulatory T cells (Tregs) serve as pivotal orchestrators of systemic immune homeostasis; consequently, their functional dysregulation is intimately associated with immune evasion in CRC.^39^ The KP actively participates in the remodelling of the CRC immune microenvironment by modulating Treg functionality. Both in vitro and in vivo studies have demonstrated that AhR activation, driven by KP metabolites, elicits profound immunosuppressive effects; specifically, it induces the differentiation of Foxp3^+^ Tregs to subvert anti‐tumour immunity while concomitantly dampening the immunogenicity of dendritic cells.^47^, ^48^ A clinical cohort study involving CRC patients revealed significant reductions in serum TRP and KYN levels, concurrently demonstrating that the expression of the forkhead box P3 (*FOXP3*) gene‐characteristically upregulated in Tregs‐positively correlated with the activation status of Tregs; furthermore, *FOXP3* expression in advanced‐stage CRC (T3/T4) was approximately 44‐fold higher than that in early‐stage CRC (T1/T2). These findings suggest that KYN may modulate Treg functionality via the regulation of *FOXP3* expression, thus orchestrating the anti‐tumour immune response and inhibiting CRC progression.^49^ Notably, while this investigation revealed marked depletions in serum TRP and KYN levels among CRC patients, KYNA concentrations remained largely unaltered, and a corresponding significant shift was lacking. This phenomenon, which seemingly contradicts canonical KP metabolic kinetics, can be elucidated from three perspectives. First, circulatory depletion primarily involves exogenous dietary TRP, which is aggressively sequestered by tumour‐upregulated amino acid transporters; conversely, endogenous TRP is efficiently recycled to sustain baseline KP metabolic flux, preventing a synchronous decline in KYNA. Second, upregulated KAT expression within the tumour microenvironment facilitates the localized conversion of KYN to KYNA. Since this KYNA predominantly mediates local immunosuppressive effects and is not extensively released into the systemic circulation, serum levels remain relatively stable. Third, KYNA can also be synthesized from IPA via a reactive oxygen species (ROS)‐dependent conversion of unstable intermediates. This alternative pathway bypasses upstream KP metabolism, thus preserving baseline KYNA levels to a certain extent.^50^

With respect to the gene regulatory mechanisms of the KP, IDO1 can accelerate CRC progression by modulating the expression of immune‐associated genes. Encoding a guanine nucleotide exchange factor (GEF), *Dock2* is frequently mutated in inflammatory bowel disease (IBD)‐associated colorectal cancer and predominantly performs immunomodulatory functions. By generating genetically engineered murine models of IBD‐associated colorectal cancer, Churchhouse AMD et al. reported that *Dock2* knockout mice exhibited altered expression profiles of interferon‐γ (IFNγ)‐associated genes, characterized by increased tryptophan metabolism and upregulated IDO1 expression. Conversely, pharmacological inhibition of IDO1 delayed tumorigenesis in *Dock2*‐deficient mice, further substantiating that targeting IDO1 can effectively impede CRC progression.^51^ Caffeine suppresses IDO1 expression via the KLF4‐COL12A1‐MAPK‐IDO1 axis, leading to reduced kynurenine production and ameliorating CD8^+^ T cell exhaustion, thus increasing the therapeutic efficacy of anti‐PD‐1 immunotherapy in colorectal cancer.^36^

#### 3.1.2 Tumour‐suppressive effects and underlying mechanisms of the KP

Colitis‐associated colorectal cancer (CAC), a subtype of colorectal cancer associated with inflammatory bowel disease (IBD), is fundamentally driven by the ‘inflammation–dysplasia’ sequence during colitis‐associated carcinogenesis. The azoxymethane‐dextran sulphate sodium (AOM‐DSS) model, which effectively recapitulates this pathological progression, has been established as a classic experimental paradigm for studying CAC and CRC.^52^ Research has revealed that KP exerts pivotal regulatory effects across different stages of colitis‐associated carcinogenesis. During the early stages of CAC, the natural compound trilobatin activates AhR and increases the production of xanthurenic acid, a downstream KP metabolite; this cascade suppresses intestinal inflammation and restores barrier function, highlighting the tumour‐suppressive potential of KP in early‐stage CAC.^53^ In advanced stages of CAC, Wumei Wan (WMW) has been shown to inhibit the conversion of tryptophan to kynurenine and ameliorate the tumour immune microenvironment, subsequently hindering CRC progression.^54^ Collectively, these findings indicate that in the mid‐to‐late tumour microenvironment of CAC, the persistently activated KP drives pro‐tumorigenic effects, a phenomenon that contrasts sharply with its anti‐inflammatory properties during the early phases of the inflammation‐to‐cancer transition, thus corroborating the dualistic role of the KP in CRC.

Furthermore, sirtuin 3 (SIRT3), an NAD^+^‐dependent enzyme in intestinal epithelial cells (IECs), is indispensable for T‐cell differentiation; SIRT3 deficiency exacerbates the production of the pro‐inflammatory cytokine IL‐1β and promotes the differentiation of local T cells and cytotoxic T lymphocytes, thus accelerating colorectal cancer progression; conversely, QA, a downstream metabolite of the KP, can serve as a substrate for de novo NAD^+^ synthesis within IECs to replenish the intracellular NAD^+^ pool depleted by SIRT3 deficiency, thus orchestrating the functions of IECs and T cells to suppress CRC development, highlighting the potential regulatory and therapeutic value of the QA‐NAD^+^ axis in colorectal cancer.^55^

In addition to these mechanisms, the KP plays a pivotal role in driving crucial biological processes in CRC, including immune evasion, metabolic reprogramming, and genomic instability.

With respect to immune evasion, the KP promotes an immunosuppressive microenvironment via multiple mechanisms. Brandacher et al. demonstrated that robust IDO1 expression in CRC cells reduces the infiltration of CD3^+^ T cells and facilitates liver metastasis by inducing local TRP depletion and generating pro‐apoptotic tryptophan metabolites, such as KYN and 3‐HK; these findings confirm that IDO1 is a central regulator that mediates tumour immune evasion in CRC.^56^ Furthermore, studies have validated that high IDO1 expression at the invasive front is an independent adverse prognostic factor in CRC and is correlated significantly with decreased overall survival and shortened metastasis‐free survival.^57^

In the context of metabolic reprogramming, Lee et al. revealed that *APC* loss activates the TCF4/β‐catenin signalling axis to upregulate TDO2 expression; subsequently synthesized KYN activates AhR; this in turn upregulates the expression of glycolysis‐associated genes, such as *SLC2A1* and *HK2*, to increase glycolytic flux, ultimately promoting tumour cell proliferation.^58^ The marked upregulation of IDO1 in CRC activates the KYN‐AhR pathway, which, synergistically with dysregulated cholesterol metabolism, drives an immunosuppressive microenvironment and metabolic reprogramming; notably, nanoparticles co‐delivering simvastatin and kynureninase can deplete intratumoral kynurenine and cholesterol to abrogate KYN‐AhR signalling, thus remodelling the immune microenvironment and potentiating the anti‐tumour efficacy of the PD‐1 blockade.^59^

With respect to genomic instability, studies have indicated that the downstream kynurenine metabolites 3‐HAA and 3‐HK generate hydrogen peroxide in the presence of Mn(II) or Cu(II) metal ions, inducing both single‐ and double‐strand DNA breaks and precipitating severe DNA damage.^60^ In glioma models, Bostian et al. demonstrated that TDO2‐derived KYN activates AhR to upregulate the expression of the error‐prone DNA polymerase *hpolκ*, culminating in replication stress and chromosomal damage; this finding unequivocally establishes that the KP can compromise genomic stability.^61^ Accordingly, we hypothesize that the KP possesses a latent capacity to induce DNA damage and drive genomic instability in colorectal cancer, although direct in vitro and in vivo experimental evidence specific to CRC is lacking.

Although the stage‐dependent dual regulatory mechanisms of the KP in CRC have been corroborated in both cellular and animal models, the inherent complexity of metabolic networks involving diverse downstream metabolites, as well as their tissue‐specific expression profiles, remains to be comprehensively validated by robust clinical studies.

#### 3.1.3 Local and systemic effects of the KP

On the basis of metabolic sources, anatomical sites of action, and the resulting biological effects, the regulatory effect of the KP on CRC can be dichotomized into localized effects within the tumour microenvironment and systemic immunological effects. Localized effects predominantly occur within the immediate vicinity of the tumour. Substantial evidence indicates that colonic epithelial cells, tumour cells, and immune cells can express IDO1 to synthesize copious amounts of the immunosuppressive metabolite KYN directly at the tumour site, thus modulating the local microenvironment via two principal axes: primarily, KYN functions as an endogenous AhR ligand to directly drive Treg differentiation^62^; secondarily, IDO‐mediated TRP depletion within the local tumour milieu activates the amino acid‐sensing GCN2 stress pathway in effector T cells, precipitating both proliferative arrest and functional energy. These two pathways operate synergistically to induce profound immunosuppression, effectively promoting a localized immunosuppressive tumour microenvironment.^63^

Conversely, systemic effects are jointly orchestrated by the liver, tumour‐draining lymph nodes, and distal immune organs. Hepatic expression of TDO facilitates the synthesis of KYN and its subsequent release into the peripheral circulation^64^; moreover, plasmacytoid dendritic cells (pDCs) residing in tumour‐draining lymph nodes express IDO1 to directly activate mature Tregs and sustain their suppressive capabilities, thus propagating localized immunosuppressive signals systemically.^65^ Similarly, CD169^+^ macrophages within the splenic marginal zone rely on IDO to establish systemic immune tolerance.^66^ Clinical observations indicate that plasma KYN concentrations and the KYN/TRP ratio serve as robust barometers of systemic tryptophan metabolism and are significantly correlated with long‐term survival and prognosis in CRC patients.^67^ Crucially, these localized and systemic effects are not strictly decoupled from one another: KYN synthesized locally at the tumour site can drain into the liver via the portal vein, thus increasing metabolite concentrations within the peripheral circulation. Reciprocally, circulating KYN can infiltrate the tumour stroma, acting in synergy with local IDO1‐derived products to further fortify the immunosuppressive microenvironment. Current literature often fails to explicitly delineate between the localized and systemic effects of the KP. Thus, future investigations leveraging tissue‐specific gene knockout, advanced metabolomics, and isotope tracing are warranted to meticulously elucidate their distinct functions and interactive regulatory networks.

### 3.2 Clinical Value of Kynurenine Metabolites in the Diagnosis and Prognostic Evaluation of Colorectal Cancer

As the primary branch of tryptophan metabolism, the KP encompasses key metabolites and rate‐limiting enzymes whose levels and activities are closely linked to disease progression. Consequently, these components are recognized as potential biomarkers for auxiliary diagnosis, clinical assessment and prognostic stratification in CRC.

Numerous clinical studies have established that plasma metabolites of the KP serve as effective discriminators between CRC patients and healthy controls. Sun XZ et al. demonstrated that, relative to healthy cohorts, CRC patients exhibit significantly decreased plasma TRP levels and a marked increase in the KYN/TRP ratio. Moreover, further analysis revealed that plasma KYN levels in CRC patients lacking tumour‐infiltrating lymphocytes (TILs) were substantially higher than those in their TIL‐positive counterparts. These findings suggest that plasma TRP concentrations and KYN/TRP ratios are correlated with CRC pathogenesis and the tumour immune microenvironment and potentially serve as both auxiliary diagnostic biomarkers and indicators for assessing tumour immunological activity.^68^ This perspective has been further corroborated by in vivo evidence: Li W et al. reported that Ganoderma lucidum polysaccharide (GLP) suppresses CRC tumour growth and significantly reduces serum KYN/TRP ratios in CRC‐bearing mice, further supporting the feasibility of using the KYN/TRP ratio as a biomarker for monitoring CRC.^69^

Furthermore, owing to the distinct advantage of non‐invasive acquisition, faecal KP metabolites similarly exhibit substantial diagnostic value for CRC. A clinical investigation comparing faecal samples from CRC patients and healthy controls (HCs) revealed that both KYN concentrations and KYN/TRP ratios were significantly elevated in the CRC cohort. Furthermore, IDO1 expression levels in intestinal tissues were positively correlated with the faecal KYN/TRP ratio, suggesting that aberrant IDO1 overexpression may drive the conversion of TRP to KYN and contribute to the pathological progression of CRC. Faecal KYN‐associated markers can indirectly reflect intestinal IDO1 activity and tryptophan metabolic status, establishing faeces as a viable sample source for the non‐invasive diagnosis of CRC.^70^

Furthermore, research has indicated that elevated IDO1 expression in CRC tissues is correlated with abnormal faecal and plasma KYN profiles and is significantly associated with decreased overall survival in CRC patients. Substantial evidence confirms that aberrant IDO overexpression is linked to unfavourable prognoses in multiple malignancies, including CRC and gastric cancer, as well as to chemoresistance and a compromised efficacy of immunotherapy.^71^, ^72^ For instance, a multicentre prospective study involving 2102 CRC patients across six cohorts measured preoperative circulating kynurenine metabolites and employed Cox proportional hazards regression to evaluate their association with all‐cause mortality. The results indicated that 290 patients (13.8%) died during a median follow‐up of 3.2 years; specifically, elevated circulating levels of TRP, XA and picolinic acid (PA) were significantly associated with a reduced risk of all‐cause mortality, whereas increased 3‐HK and QA levels correlated with increased mortality risk. Notably, an elevated KYN/TRP ratio, a hallmark of cell‐mediated immune activation, was found to be associated with an increased risk of mortality.^67^ By investigating a large‐scale cohort, this study confirms that circulating kynurenine pathway metabolites are robust prognostic indicators for CRC patients, providing novel clinical evidence for prognostic risk stratification. Subsequent analysis of this cohort by Victoria Damerell et al. revealed that the protective efficacy of XA is strongly dose dependent; specifically, each doubling of the XA concentration within the peripheral circulation corresponds to a 26% reduction in all‐cause mortality risk among CRC patients, a protective benefit that is particularly pronounced in individuals with stage II and III disease.^67^

Recent studies have shown that kynurenine derived from the peritumoral adipose tissue of patients with colorectal cancer can be metabolized into 3‐HK by tumour cells; the latter subsequently binds to nuclear receptor coactivator 4 (NCOA4) to suppress ferritinophagy and attenuate the release of free iron, thus mediating ferroptosis resistance within tumour cells. Furthermore, pharmacological inhibition of KP can restore sensitivity to ferroptosis, consequently potentiating the therapeutic efficacy of anti‐PD‐1 blockade.^73^

### 3.3 KP‐targeted therapeutic strategies for colorectal cancer intervention

#### 3.3.1 Targeting pivotal enzymes to modulate the KP for CRC therapy

Rate‐limiting enzymes of the KP, including IDO1/2 and TDO, serve as critical regulators of metabolic flux. Notably, IDO, the primary rate‐limiting enzyme in the initial step of the KP, has been identified as a key factor intimately linked to CRC immunotherapy. For instance, clinical evidence has demonstrated that IDO1 is significantly upregulated in CRC patients, with its expression levels positively correlated with immune infiltration scores. These findings indicate that IDO1 may drive CRC progression by modulating immune cell infiltration, thus providing a robust theoretical foundation for the development of IDO1 small‐molecule inhibitors or combination therapeutic strategies.^74^ To date, multiple classes of IDO1 inhibitors have progressed through preclinical evaluation into early‐phase clinical trials, demonstrating promising preliminary therapeutic potential.

To address the distinct immune phenotypes of CRC, several studies have investigated the therapeutic potential of combining IDO1 inhibitors with immune checkpoint inhibitors (ICIs) in microsatellite stable (MSS) CRC; for instance, co‐administration of the IDO1 inhibitor epacadostat and the PD‐1 inhibitor InVivoMAb in MSS CRC significantly slows tumour growth while concurrently enhancing the infiltration of pro‐inflammatory macrophages and CD8^+^ T cells within the tumour microenvironment. Furthermore, IDO1 knockdown upregulates the JAK2‐STAT3‐IL6 signalling axis, driving macrophage polarization towards a pro‐inflammatory phenotype and augmenting their infiltration, which synergistically potentiates the therapeutic efficacy of PD‐1 blockade. These findings further underscore the profound potential of dual IDO1 and ICI blockade in overcoming immunotherapy resistance in MSS CRC.^74^ Beyond combinations with ICIs, strategies pairing IDO1 inhibitors with other targeted therapeutics are currently under active investigation. For example, studies have revealed that the expression of both histone deacetylase (HDAC) and IDO1‐key orchestrators of immune responses within the TME‐is notably attenuated in MSS/pMMR CRC. Building upon this rationale, researchers engineered nanomedicines (NP‐I/P) co‐loaded with epacadostat (an IDO1 inhibitor) and panobinostat (an HDAC inhibitor); the findings revealed that NP‐I/P significantly reversed the immunosuppressive state by inducing immunogenic cell death (ICD), increasing CD8^+^ T‐cell infiltration, and concurrently reducing the populations of Tregs and tumour‐associated macrophages; moreover, across patient‐derived xenograft (PDX) and organoid models, NP‐I/P consistently induced ICD and beneficially modulated the immune microenvironment.^75^

Furthermore, post‐translational modifications of IDO1 play a critical role in modulating tumour immunity. For instance, in a subcutaneous CRC mouse model, Shi D et al. demonstrated that knockdown of the proteasome‐associated deubiquitinase USP14 reduces IDO1 expression levels, thus reversing the exhaustion of cytotoxic T cells and sensitizing mice to PD‐1 blockade therapy. Mechanistic investigations further revealed that USP14 knockdown did not significantly alter AhR activation induced by the IDO1 inhibitor epacadostat, indicating that USP14 functions by modulating the post‐translational stability of IDO1 rather than interfering with the AhR signalling pathway. Thus, USP14 represents a potential therapeutic target for the indirect inhibition of IDO1.^71^

In preliminary in vivo and in vitro studies, IDO inhibitors have demonstrated promising preclinical anti‐tumour efficacy. However, a phase Ia monotherapy trial (NCT02048709) encompassing diverse advanced solid tumours‐with colorectal cancer constituting a major subgroup‐revealed that while the IDO1 inhibitor navoximod significantly depleted plasma kynurenine, its monotherapeutic anti‐tumour activity remained limited, with all colorectal cancer patients experiencing disease progression.^76^ A subsequent phase Ib combination trial (NCT02471846) further evaluated the efficacy of navoximod combined with the PD‐1 inhibitor atezolizumab; the results indicated that although plasma kynurenine concentrations decreased dose‐dependently with navoximod escalation, objective responses were achieved in only a small subset of patients.^77^ In contrast, combined IDO and PD‐1 blockade regimens have yielded remarkable clinical benefits in other malignancies: the IDO/PD‐1‐targeted vaccine IO102/IO103, administered in combination with the PD‐1 inhibitor nivolumab, demonstrated exceptional efficacy in a melanoma clinical trial (NCT03047928), achieving an objective response rate (ORR) of 80%.^78^ Furthermore, the IDO inhibitor BMS‐986205, when combined with radiotherapy and nivolumab for the treatment of glioblastoma (NCT04047706), significantly prolonged patient survival. Currently, relevant clinical trials focused on colorectal cancer remain exceedingly scarce; nonetheless, given the promising therapeutic potential of combining IDO and PD‐1 inhibition in other solid tumours, the applicability and efficacy of this combinatorial immunotherapeutic strategy in colorectal cancer warrant further rigorous investigation.^79^

Nevertheless, the clinical translation of IDO1 inhibitors has encountered significant hurdles, with several trials reporting suboptimal efficacy for both monotherapy and combination approaches, possibly owing to potential off‐target effects. For instance, a recent study revealed that compared with either IDO1 or TDO2, interleukin‐4‐induced gene 1 (IL4I1) is more strongly correlated with AhR activity. Furthermore, indole‐3‐propionic acid (I3P) has been shown to mediate IL4I1‐driven AhR activation, thus promoting the progression of haematological malignancies.^80^ Because IDO inhibitors fail to suppress IL4I1, AhR‐mediated immunosuppression may persist, potentially compromising therapeutic outcomes. A phase III clinical trial in advanced melanoma reported disappointing results for the combination of epacadostat and pembrolizumab, providing a possible explanation for the setbacks observed in IDO1 inhibitor clinical development.^81^ Moreover, the inter‐patient heterogeneity of IDO1 expression within the same tumour type suggests that IDO1 inhibitors may be more effective in CRC subgroups characterized by high IDO1 expression or pre‐existing immunotherapy resistance. Such variability underscores the need for patient stratification on the basis of IDO1 expression levels in future clinical practice to identify responsive cohorts and enhance precision medicine.^82^

Furthermore, although current investigations have reported that tumour‐suppressive effects are mediated by IDO1 inhibitors, robust evidence confirming direct inhibitor engagement with intratumoral targets is lacking. To definitively attribute anti‐tumour efficacy to the specific blockade of IDO1 rather than potential off‐target effects, studies must supplement evaluations of tumour‐associated phenotypic alterations with direct pharmacodynamic evidence. For instance, this could involve demonstrating a profound reduction in the local KYN/TRP ratio within the tumour microenvironment, showing transcriptional suppression of AhR downstream target genes, or providing explicit data regarding IDO1 enzymatic activity and target occupancy rates. The absence of direct in situ target validation may fundamentally explain why several IDO1 inhibitors have failed to meet efficacy expectations in clinical trials.

In addition to IDO, KMO and KYNU also perform critical functions within the KP. For example, evidence indicates that KMO expression is markedly elevated in colorectal cancer tissues compared with healthy mucosa and polyps. Furthermore, CRC patients harbouring high KMO expression face an exacerbated risk of metastasis and poor long‐term survival prognoses. In vitro experiments have demonstrated that KMO knockout attenuates the expression of the colorectal cancer stem cell marker CD44 and diminishes the migratory and invasive capacities of CRC cells, thus corroborating that KMO drives CRC tumorigenesis, invasion and metastasis.^83^

In addition, researchers reported that compared with its human counterpart, a PEGylated KYNU (PEG‐KYNase) derived from *Pseudomonas fluorescens* exhibits substantially enhanced catalytic activity toward KYN. Subsequent in vivo evaluations revealed that PEG‐KYNase efficiently depleted KYN within the tumour microenvironment to reverse IDO1/TDO‐mediated immunosuppression and increase the intratumoral infiltration and functionality of CD8^+^ T cells, ultimately prolonging the survival of tumour‐bearing mice. When administered in combination with PD‐1 blockade or tumour vaccines, it elicits synergistic anti‐tumour effects that further potentiate overall therapeutic efficacy.^84^ Consequently, the limited monotherapeutic efficacy of IDO1 inhibitors, the emergence of IL4I1 bypass compensation, and the lack of direct verification regarding tumour target engagement represent major bottlenecks in current clinical translation; dual‐target inhibitors and combinatorial drug delivery strategies thus warrant rigorous future investigations.

#### 3.3.2 Natural Products as Modulators of the KP for Colorectal Cancer Therapy

Natural products exert therapeutic effects on CRC by modulating key enzymes, metabolites, or receptors within the KP. Trilobatin, a natural compound, targets the KP via a dual mechanism to exert anticancer effects during the early stages of colitis‐associated carcinogenesis. First, trilobatin acts as a direct and specific ligand for AhR, thus triggering the AhR signalling cascade. Second, it maintains gut microbiota homeostasis and promotes the generation of xanthurenic acid (XA), a downstream metabolite of the KP. XA subsequently upregulates AhR expression, thus alleviating the symptoms of ulcerative colitis. These findings suggest that trilobatin‐mediated targeting of the KP during early‐stage CAC can mitigate colitis symptoms and hinder colitis‐associated carcinogenesis.^53^ Similarly, the tumour‐suppressive effects of Ganoderma lucidum polysaccharide (GLP) on CRC are contingent upon the modulation of key enzymes in the KP. GLP downregulates IDO1 expression to suppress TRP‐to‐KYN conversion while simultaneously increasing the infiltration of antitumour CD8^+^ T cells to inhibit CRC growth. In vivo studies further demonstrated that combining GLP with PD‐1 blockade significantly increased Th1 cell infiltration both locally and peripherally, leading to synergistic immunotherapeutic effects. This combination offers a promising strategy for enhancing the efficacy of CRC immunotherapy.^69^

Furthermore, emerging evidence has demonstrated that Wumei Wan (WMW), a traditional Chinese medicine formula, suppresses CRC progression by targeting the KP. In AOM/DSS‐induced CRC mouse models, WMW inhibits IDO1 activity, thus reducing the catabolic conversion of tryptophan into KYN and subsequently dampening the aberrant activation of the ‘IDO1‐KYN‐AhR’ signalling axis. Metabolomic profiling further corroborated that WMW treatment significantly decreased the levels of downstream KP metabolites, including kynurenic acid, 3‐HAA and QA, within tumour tissues. Moreover, the marked reduction in the KYN/TRP ratio indicates that WMW effectively restricts metabolic flux through the tryptophan‐kynurenine pathway.^54^ These findings suggest that during the advanced stages of CAC, WMW remodels the tumour immune microenvironment by targeting the ‘IDO1‐KYN‐AhR’ axis, providing novel metabolic targets and therapeutic strategies for CAC.

#### 3.3.3 Therapeutic Targeting of the Gut Microbiota to Modulate the KP in CRC

KP is involved primarily in a host‐intrinsic metabolic cascade. Within the intestinal tract, IDO1 serves as the predominant rate‐limiting enzyme for this pathway, whereas TDO and IDO2 are essentially unexpressed in intestinal tissues.^85^ However, accumulating evidence firmly establishes that the gut microbiota also actively participates in the regulation of the KP: on the one hand, it indirectly modulates pathway activity by orchestrating host inflammatory responses and subsequently altering rate‐limiting enzyme expression; on the other hand, a select minority of bacterial taxa encode orthologs of key host enzymes, with the inherent capacity for de novo kynurenine biosynthesis.

Current research indicates that the gut microbiota more pervasively regulates the KP by influencing the expression of inflammatory cytokines, which upregulate host IDO1 expression and subsequently drive aberrant KP activation. Lipopolysaccharide (LPS) released by pro‐tumorigenic Proteobacteria can trigger immune responses in colonocytes and upregulate IDO1 levels, skewing tryptophan metabolism toward the KP. This ultimately results in localized tryptophan depletion and massive accumulation of the immunosuppressive metabolite KYN.^86^ Furthermore, the gut microbiota can profoundly influence the KP through mechanisms such as direct translocation and metabolic modulation of immune cells. As a commensal bacterium capable of translocating to mesenteric tissues, *Achromobacter pulmonis* has been shown to activate the TLR4 signalling pathway in macrophages, upregulate IDO1 activity, and drive the local metabolic shunting of tryptophan towards kynurenine, precipitating a substantial accumulation of immunosuppressive KYN.^20^ *Fusobacterium nucleatum* is markedly enriched in colorectal cancer. Xue Y et al. reported that *Fusobacterium nucleatum* can invade macrophages and activate the TLR4/MyD88‐NF‐κB signalling axis via its surface adhesin FadA and LPS; simultaneously, it significantly upregulates macrophage IDO1 expression via a TNF‐α‐dependent inflammatory pathway, culminating in intestinal tryptophan exhaustion and massive KYN accumulation. Notably, although the IDO‐mediated microenvironment‐characterized by low tryptophan levels and high KYN levels‐can restrict the intracellular proliferation of *Fusobacterium nucleatum* within macrophages, robust IDO expression concomitantly suppresses peripheral lymphocyte function. This shields *Fusobacterium nucleatum*‐infected macrophages from immune clearance, exacerbating immunosuppression within the tumour microenvironment and promoting CRC progression.^87^

In stark contrast, beneficial commensal bacteria are capable of restraining hyperactivation of the KP. By utilizing an *Apc* ^Min/+^ spontaneous colorectal cancer model, Gao H et al. demonstrated that GSDMD deficiency significantly suppressed intestinal tumorigenesis. Gasdermin D (GSDMD), an inflammasome effector protein that mediates pyroptosis, is significantly upregulated in both human and murine intestinal tumours; further investigations revealed that the faecal KYN levels in *Apc* ^Min/+^ *Gsdmd* ^−/−^ mice correlate negatively with the abundance of *Lactobacillus* and that exogenous KYN supplementation actively promotes intestinal tumour development in these *Apc* ^Min/+^ *Gsdmd* ^−/−^ mice. These findings suggest that *Lactobacillus* may restrain CRC progression by attenuating systemic KYN levels, thus providing robust experimental evidence for synergistic therapeutic strategies targeting both the gut microbiota and the KP.^88^

Beyond indirectly modulating pathway activity, a select subset of the gut microbiota encodes catalytic enzymes homologous to those in the host kynurenine pathway, enabling the direct synthesis of kynurenine and its downstream metabolites. Through protein functional database alignments and in vitro culture validation, Agus A et al. reported that pathogenic Proteobacteria, such as *Pseudomonas* and *Burkholderia*, encode kynurenine formamidase (homologous to the host arylformamidase AFMID) and kynureninase (KynU) (homologous to the host KYNU); these enzymes allow them to use environmental tryptophan to synthesize KYN and 3‐HAA.^89^ We hypothesize that in the context of CRC‐associated dysbiosis, microbiota‐derived kynurenine produced by pathogenic bacteria acts synergistically with the endogenous host IDO1‐mediated pathway to increase immunosuppressive KYN levels within the tumour microenvironment. This synergy likely amplifies tumour immune evasion and promotes CRC progression, although its precise in vivo impact warrants further empirical validation.

In summary, targeting the gut microbiota can provide novel avenues for mechanistic studies and therapeutic developments in CRC by modulating the immune microenvironment and host–microbiota cross‐talk.

#### 3.3.4 KP related target gene knockout: Insights into target specific functions

To elucidate the functional specificity of the KP in CRC progression, accumulating studies have performed in vivo and in vitro validation using genetic knockdown and knockout models.

Wu et al. demonstrated that silencing IDO1 in colorectal cancer cells curtailed kynurenine production; subsequent animal experiments further corroborated that IDO1 knockdown downregulated thymocyte selection‐associated HMG box expression in tumour‐infiltrating CD8^+^ T cells, thereby reversing kynurenine‐induced T‐cell exhaustion.^90^ Similarly, research by Li et al. revealed that IDO1 silencing modulated the expression of ferroptosis‐related genes *ACSL4*, *GPX4* and *SLC7A11* and augmented the suppressive effects of ferroptosis inducers Erastin and RSL3 on cancer cell proliferation and migration, confirming that IDO1 participates in CRC progression through ferroptosis pathway regulation.^91^ Furthermore, Bishnupuri et al. discovered in an azoxymethane/dextran sodium sulphate AOM/DSS‐induced CRC mouse model that intestinal epithelial‐specific IDO1 knockout markedly reduced tumour multiplicity and volume while inhibiting proliferation and promoting apoptosis. Their findings indicate that epithelial IDO1 functions as a pivotal driver of colorectal tumorigenesis, and that IDO1‐catalyzed kynurenine metabolites activate the PI3K–Akt signalling pathway to stimulate epithelial cell proliferation and suppress apoptosis.^92^

Beyond driving tumour progression proper, the myeloid cell‐mediated IDO1–kynurenine axis also contributes to chemotherapy‐induced intestinal toxicity. In an oxaliplatin chemotherapy model using MC38 subcutaneous tumour‐bearing mice, myeloid cell‐specific IDO1 deletion substantially alleviated chemotherapy‐associated toxicities, including body weight loss, intestinal histopathological damage, and crypt cell apoptosis. Notably, this pathological injury was similarly mitigated by engineered probiotics overexpressing kynurenine aminotransferase, indicating that, in addition to intestinal epithelial cells, myeloid‐derived IDO1 exerts a critical influence on intestinal barrier homeostasis.^93^

**FIGURE 2:**
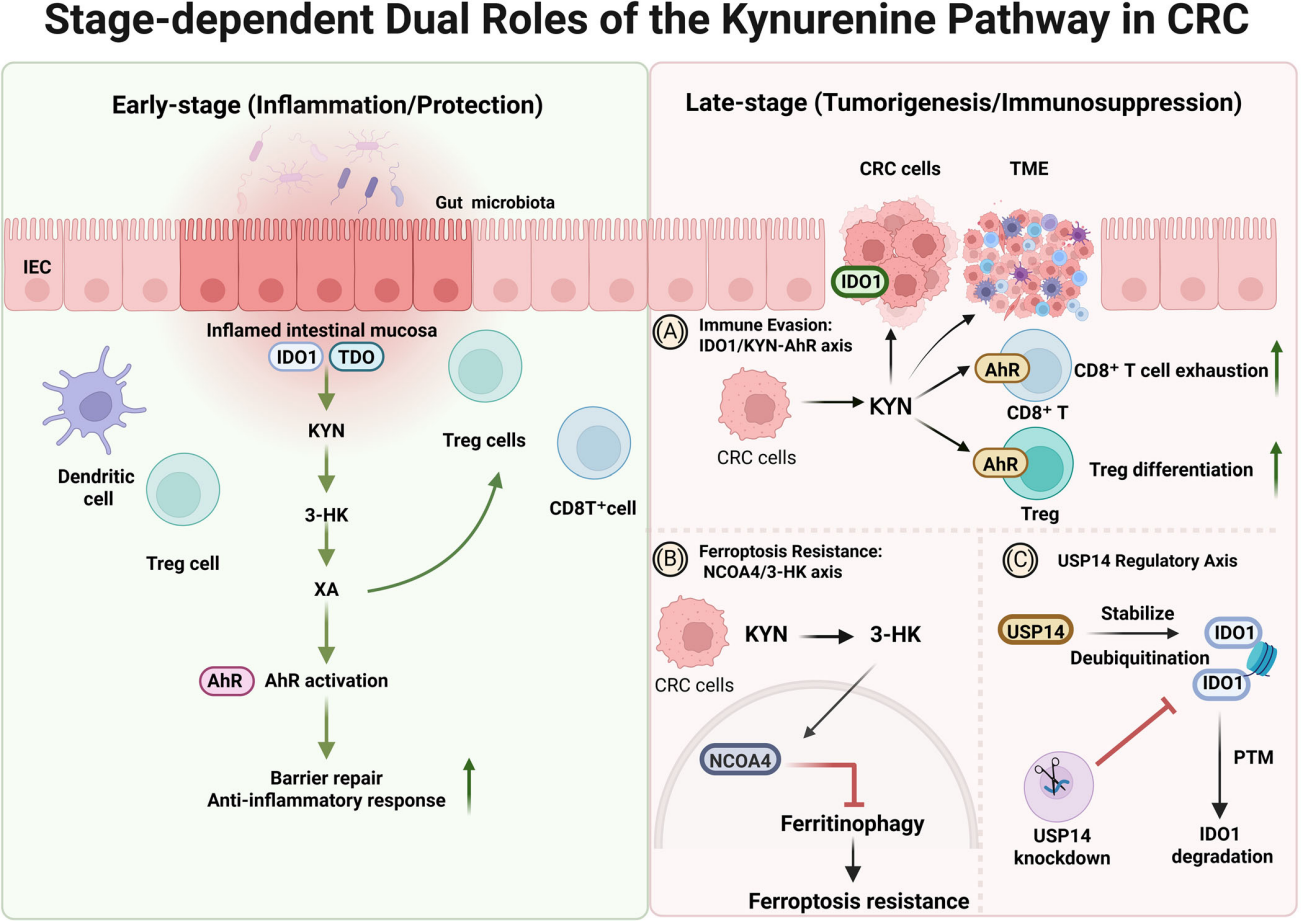
Stage‐dependent dual roles of the kynurenine pathway in CRC. The kynurenine pathway exerts context‐dependent biological effects during CRC progression. Left panel: In early‐stage inflamed intestinal mucosa, IDO1 and TDO are expressed by intestinal epithelial cells and immune cells. Metabolites generated through the KYN‐3‐HK‐XA cascade activate aryl hydrocarbon receptor (AhR), promote regulatory T‐cell (Treg) differentiation, and initiate intestinal barrier repair and anti‐inflammatory responses. Of note, 3‐HK is produced in both early and late stages; it is further metabolized into XA to confer protective effects under inflammatory conditions, while accumulating in CRC cells to mediate oncogenic functions at the tumorigenesis stage. Right panel: In late‐stage tumorigenesis, IDO1‐overexpressing CRC cells produce abundant KYN. (A) IDO1/KYN‐AhR immune‐evasion axis: accumulated KYN activates AhR, which drives CD8^+^ T cell exhaustion and Treg differentiation to construct an immunosuppressive tumour microenvironment (TME). (B) NCOA4/3‐HK axis mediating ferroptosis resistance: 3‐HK inhibits NCOA4‐dependent ferritinophagy, thereby endowing CRC cells with a ferroptosis‐resistant phenotype. (C) USP14 regulatory axis: USP14 stabilizes IDO1 via post‐translational modification (PTM). USP14 knockdown triggers IDO1 degradation, and this regulatory effect does not directly interfere with AhR signalling. IEC, intestinal epithelial cell; TME, tumour microenvironment.

Collectively, while these insights advance our understanding, experimental evidence regarding cell type‐specific in vivo knockout of the KP in CRC remains relatively scarce, and the underlying molecular mechanisms warrant further investigation through more rigorous exploration. Collectively, the functional mechanisms of kynurenine‐pathway‐related markers and the stage‐dependent regulatory network of this pathway in colorectal cancer can be found in Table 1 and Figure 2.

**TABLE 1:** Functional mechanisms of kynurenine pathway‐related markers in CRC.

Molecule	Subject/Sample	Functional Mechanism	Reference
KYN/TRP	Mouse CRC model/Serum	GLP can inhibit CRC tumour growth and significantly reduce the KYN/TRP ratio in the serum of CRC mice.	PMID:38750669
KYN/TRP	CRC patients/Serum	An elevated serum TRP/KYN ratio is associated with increased mortality risk and serves as a prognostic indicator	PMID: 39308420
KYN	Mouse CRC model/Faeces	Faecal KYN levels in Apc^Min/+^Gsdmd^−/−^mice correlate negatively with *Lactobacillus* abundance, KYN supplementation promotes CRC progression.	PMID: 39434144
3‐HK	CRC patients/Serum	Increased serum 3‐HK concentration correlates with higher all‐cause mortality risk and prognostic assessment.	PMID: 39308420
3‐HK	CRC cells	CRC cells exhibit high IDO1 expression, which reduces the number of CD3^+^ tumour‐infiltrating T cells via local TRP depletion and the production of KYN and 3‐HK.	PMID: 16489067
3‐HK	CRC cells	Peritumoral adipose tissue of CRC can synthesize 3‐HK, which binds to NCOA4 to inhibit ferritinophagy, reduce free‐iron release, and mediate ferroptosis resistance in tumour cells.	PMID: 41813885
QA	CRC cells/Mouse CRC model	QA can serve as a substrate for de novo NAD^+^ synthesis within IECs to replenish the intracellular NAD^+^ pool depleted by SIRT3 deficiency, thereby orchestrating the functions of IECs and T cells to suppress CRC development.	PMID: 41487042
QA	CRC patients/Serum	Elevated serum QA concentration is linked to increased mortality risk and aids in prognostic evaluation.	PMID: 39308420
IDO1	CRC patients/Serum	Elevated IDO1 expression is correlated with shortened overall survival and resistance to immunotherapy in patients with CRC.	PMID: 39751692
IDO1	CRC cells	Elevated IDO1 expression at the tumour invasive front serves as an independent adverse prognostic factor in CRC, showing a significant correlation with shortened overall survival and accelerated tumour metastasis.	PMID: 22108515
IDO1	CRC cells/Mouse CRC model	Highly expressed IDO1 in CRC activates the KYN‐AhR pathway and synergizes with cholesterol metabolic dysregulation, thereby driving an immunosuppressive microenvironment and metabolic reprogramming.	PMID: 41432057
IDO1	CRC cells/Mouse CRC model	IDO1 knockdown in CRC cells decreases the generation of KYN, In vivo experiments demonstrated that IDO1 knockdown downregulates the expression of thymocyte selection‐associated high mobility group box protein in tumour‐infiltrating CD8^+^T cells, thereby reversing kynurenine‐induced T cell exhaustion.	PMID: 33841677
IDO1	CRC cells	IDO1 silencing regulates the expression of ferroptosis‐related genes *ACSL4*, *GPX4*, and *SLC7A11*, thereby potentiating the suppressive effects of ferroptosis inducers Erastin and RSL3 on CRC cell proliferation and migration.	PMID: 42291098
IDO1	Mouse CRC model	Intestinal epithelial cell‐specific knockout of IDO1 significantly decreases tumour multiplicity, reduces tumour volume, and suppresses tumour cell proliferation.	PMID: 30679179
IDO1	Mouse CRC model	WMW inhibits the overactivation of the IDO1‐KYN‐AhR axis, reduces kynurenine production, and remodels the immune microenvironment to suppress CRC progression.	PMID: 40780480
IDO1	IDO1 inhibitor (epacadostat)	USP14 knockdown modulates post‐translational modifications of IDO1, enhancing CRC immunotherapy efficacy.	PMID: 36163134
IDO1	CRC patients/Serum	High IDO1 expression is associated with shortened overall survival and resistance to immunotherapy.	PMID: 39751692
IDO1	IDO1 inhibitor (navoximod)	While navoximod, an IDO1 inhibitor, significantly lowers plasma KYN, it exhibits only modest antitumour efficacy as monotherapy, with disease progression occurring across all CRC patients.	PMID: 29921320
IDO1+PD‐1	IDO1 inhibitor(epacadostat)	Combining epacadostat with a PD‐1 inhibitor InVivoMAb increases CD8^+^T cell infiltration in MSS CRC and inhibits tumour growth.	PMID: 39751692
IDO1+PD‐1	IDO1 inhibitor (navoximod)	Combination of the IDO1 inhibitor navoximod with the PD‐1 inhibitor atezolizumab enables sustained reduction of plasma KYN with increasing navoximod doses; however, only a small subset of patients achieve objective response.	PMID: 30770348
IDO1+HDAC	IDO1 inhibitor (epacadostat)	Nanomedicine co‐loaded with epacadostat and panobinostat induces immunogenic cell death and enhances CRC immunotherapy efficacy.	PMID: 39676171
KMO	CRC patients /tumour tissues	Compared with healthy tissues and polyps, KMO expression levels are significantly elevated in colorectal cancer tissues. Furthermore, patients with high KMO expression exhibit an increased risk of tumour metastasis and a poorer long‐term survival prognosis.	PMID: 33937026

Abbreviations: 3‐HK, 3‐hydroxykynurenine; ACSL4, acyl‐CoA synthetase long‐chain family member 4; AhR, aryl hydrocarbon receptor; CRC, colorectal cancer; GLP, ganoderma lucidum polysaccharide; GPX4, glutathione peroxidase 4; GSDMD, gasdermin D; IDO1, indoleamine‐2,3‐dioxygenase 1; IEC, intestinal epithelial cell; KYN, kynurenine; MSS, microsatellite‐stable; NAD^+^, nicotinamide adenine dinucleotide; NCOA4, nuclear receptor coactivator 4; PD‐1, programmed cell death protein 1; QA, quinolinic acid; SLC7A11, solute carrier family 7 member 11; TRP, tryptophan; USP14, ubiquitin‐specific protease 14; WMW, Wumei Wan.

## 4 THE SEROTONIN PATHWAY IN COLORECTAL CANCER

Serotonin is a monoamine neurotransmitter that performs critical biological functions systemically owing to the widespread distribution of its receptors. 5‐hydroxytryptamine receptors (5‐HTRs) bind to serotonin to activate downstream signalling cascades, actively participating in physiological processes such as lipid metabolism and insulin secretion, which consequently affect normal physiological homeostasis and disease pathogenesis.^67^, ^94^, ^95^ In addition to its classical roles in emotional regulation and cognitive maintenance within the central nervous system, serotonin plays a pivotal role in modulating osteoblast activity and inhibiting ferroptosis in tumour cells.^96^, ^97^, ^98^, ^99^ Furthermore, acting as an essential precursor for melatonin synthesis, serotonin is strongly involved in the regulation of sleep and circadian rhythms.^100^

Although the serotonin pathway accounts for merely 1%–2% of overall tryptophan metabolism, it plays an indispensable role in the oncogenesis and progression of CRC. Recent evidence indicates that the serotonin pathway exerts a complex, dualistic influence on CRC: it inherently possesses tumour‐suppressive properties, yet it can also drive pro‐tumorigenic effects during specific stages of the disease. The underlying mechanisms are multifaceted and include the regulation of tumour cell proliferation and cancer stem cell functionality, the remodelling of the immune microenvironment, and the aberrant activation of crucial signalling cascades.

### 4.1 Pro‐tumorigenic Effects of the Serotonin Pathway in CRC

As early as 1978, Tutton PJ et al. first demonstrated that serotonin activates 5‐HTRs to increase the mitotic rate of CRC cells, thus directly promoting tumour cell proliferation.^101^ Recent studies have further elucidated that serotonin drives the oncogenesis and progression of CRC through multi‐target and multi‐pathway mechanisms, encompassing both receptor‐dependent and receptor‐independent mechanisms.

Serotonin regulates the stemness of CRC stem cells. Colorectal cancer stem cells (CSCs) constitute a subpopulation of tumour cells characterized by self‐renewal and differentiation capacities and play critical roles in tumorigenesis, metastasis, drug resistance and recurrence. Through clinical investigations, Zhu P et al. reported that serotonin levels are significantly elevated in advanced and metastatic CRC lesions; mechanistic studies further corroborated that high levels of serotonin can bind to the abundantly expressed HTR1B/1D/1F receptors on CSCs, activating the Wnt/β‐catenin signalling cascade to promote CSC self‐renewal. Furthermore, serotonin increases the CSC ratio and enhances both sphere formation and metastatic capabilities, suggesting a positive correlation between systemic serotonin levels and CRC severity.^102^

Serotonin activates inflammatory responses in the tumour microenvironment. The pro‐tumorigenic effect of serotonin is closely associated with the assembly and activation of inflammasomes in immune cells. Li T et al. reported that the levels of both serotonin and TPH1 are significantly upregulated in the tumour tissues of CRC patients and murine CRC models, as well as in CRC cell lines; elevated serotonin enhances the activation of the NLRP3 inflammasome in THP‐1 monocytes. Subsequent in vivo experiments verified that *NLRP3* knockout or intervention with the TPH1‐specific inhibitor pCPA significantly suppresses AOM/DSS‐induced CRC tumour growth and metastasis, indicating that the serotonin‐NLRP3 axis may serve as a crucial nodal point in CRC regulation.^103^

Serotonin mediates epithelial–mesenchymal transition (EMT) and receptor‐dependent regulation. Li T et al. reported that HTR2B is highly expressed in CRC tumour tissues, where it induces CREB1 phosphorylation by activating the Akt/mTOR signalling pathway; phosphorylated CREB1 subsequently binds to the promoter of E‐box binding zinc finger protein 1 (ZEB1) to promote its transcription, thus inducing EMT and enhancing tumour cell migration and invasion. Notably, the HTR2B‐specific antagonist RS127445 significantly ameliorated metastasis and reversed the EMT process in murine models.^104^ Lee JY et al. further confirmed that HTR2B inhibition blocks the sustained activation of the ERK signalling cascade, thus suppressing the proliferative and migratory capacities of CRC cells.^105^ In addition, Ling T et al. demonstrated that depleting serotonin via TPH1 silencing or the TPH1 inhibitor L‐p‐chlorophenylalanine (L‐PCPA) significantly restrains CRC growth, confirming that serotonin promotes CRC progression through classical serotonin receptor pathways.^106^

Receptor‐independent regulation by serotonin: serotonylation. While previous studies predominantly focused on the interactions between serotonin and 5‐HTRs, recent research has revealed that serotonin can achieve receptor‐independent functional regulation through the formation of stable covalent bonds with intracellular proteins, a process termed ‘serotonylation’. For example, the serotonylation of histone H3 glutamine 5 (H3Q5ser) in CRC cells and cancer‐associated fibroblasts (CAFs) induces the phenotypic transition of CAFs into an inflammatory‐like subtype (iCAFs), thus increasing CRC cell proliferation, invasion, and macrophage polarization. Conversely, knocking out the serotonin transporter SLC22A3 or inhibiting transglutaminase 2 (TGM2) decreases H3Q5ser levels and reverses the phenotypic transition of CAFs, suggesting that targeting serotonylation holds promise as a novel therapeutic strategy for CRC.^106^ This finding also highlights that, in addition to the extensively studied tumour and immune cells, the regulatory mechanisms of stromal cells such as CAFs in CRC warrant significant attention.

Furthermore, Yu H et al. reported that serotonin enters cells via the serotonin transporter (SERT) and undergoes transglutaminase 2 (TGM2)‐mediated serotonylation of Ras homolog family member A (RhoA). This activity activates the RhoA/Rho‐associated kinases (ROCKs)/Yes‐associated protein (YAP) signalling cascade, upregulating YAP expression in CRC cells to promote CRC progression. Clinical data indicate that serum serotonin levels are significantly higher in CRC patients than in healthy individuals and are particularly elevated in patients with multiple metastatic CRC, further corroborating the potential regulatory role of serotonin in the progression and metastasis of CRC.^107^

The gut microbiota also plays a crucial role in the serotonin pathway. On the one hand, a diverse array of bacteria, including *Lactobacillus*, *Lactococcus*, *Escherichia coli*, *Streptococcus* and *Klebsiella*, can express tryptophan synthase to produce serotonin.^108^ However, microbiota‐derived serotonin accounts for only 10% of total peripheral serotonin, indicating that the primary mechanism through which the microbiota regulates serotonin is through the activation of host biosynthetic pathways. Spore‐forming consortia in the gut, primarily dominated by *Clostridium*, can generate metabolites such as deoxycholic acid and butyrate; these metabolites act on colonic enterochromaffin cells to upregulate TPH1 expression, thus promoting host serotonin synthesis. Animal studies have revealed that germ‐free mice exhibit significantly reduced serotonin levels in both the colon and circulation, accompanied by massive TRP accumulation; transplantation of spore‐forming consortia restores colonic TPH1 expression and serotonin content, an effect that is completely abrogated by antibiotic‐mediated depletion of the gut microbiota.^109^

In addition, compared with their conventionally raised counterparts, mice living in a germ‐free environment exhibit significantly reduced systemic serotonin levels and colonic TPH expression.^110^

Systemic levels of melatonin, a downstream metabolite of the serotonin pathway, are similarly modulated by the gut microbiota. Studies have shown that metronidazole‐treated mice and germ‐free mice exhibit decreased serum melatonin levels but elevated local colonic melatonin concentrations, suggesting that intestinal microbes actively participate in regulating host melatonin biosynthesis^111^ Furthermore, melatonin has been proven to maintain the integrity of the intestinal barrier and modulate the community structure of the gut microbiota.^112^

### 4.2 Inhibitory roles of the Serotonin pathway in CRC progression

Although serotonin predominantly exerts pro‐tumorigenic effects in CRC, recent studies have revealed that its functions are highly disease‐stage specific and that it exhibits tumour‐suppressive properties during the early stages of CRC. This dualistic role hinges primarily on the dynamic switching of signalling cascades and the spatial remodelling of the immune microenvironment. 5‐HT2B serves as a pivotal serotonin receptor that is predominantly expressed in IEC. By engineering mutant mice with intestinal epithelial‐specific knockout of *Htr2b*, Mao L et al. reported that during the initiation phase of colitis‐associated cancer (CAC), the serotonin/5‐HT2B axis mitigates inflammation and epithelial damage via the activation of SMAD‐dependent TGF‐β signalling, thus suppressing CAC tumorigenesis. Conversely, during the advanced stages of CAC, the serotonin/5‐HT2B axis activates Akt through a SMAD‐independent TGF‐β signalling pathway, which actively promotes tumour cell proliferation, migration and angiogenesis. At this advanced stage, *Htr2b* deletion significantly diminished tumour burden and concomitantly reduced phosphorylated Akt levels in murine tumour tissues, indicating that serotonin/5‐HT2B exerts pro‐tumorigenic effects in late‐stage CAC by modulating Akt activity. This study unequivocally defines the stage‐dependent functional transition of the serotonin/HTR2B/TGF‐β signalling axis in CAC, furnishing critical evidence for the dualistic role of the serotonin pathway in CRC.^113^ Similarly, Sakita JY et al. demonstrated that in *TPH1*‐knockout CRC subcutaneous models and AOM/DSS‐induced colon cancer models, a reduction in systemic serotonin levels successfully inhibited CRC progression, reaffirming the pro‐tumorigenic capacity of serotonin; however, further mechanistic investigations revealed that this serotonin depletion exacerbated colonic DNA damage, suggesting that serotonin intrinsically promotes DNA repair during early‐stage CRC to exert a protective, tumour‐suppressive effect.^114^ Wen et al. demonstrated that IHCH‐8110, a non‐blood‐brain barrier‐penetrating agonist, stimulates enteric glial cells (EGCs) to secrete CXCL10 and IL‐18. CXCL10 recruits CD8^+^ T cells to infiltrate the tumour microenvironment, while IL‐18 enhances their cytotoxic activity. Notably, the tumour‐suppressive effect was completely abolished following EGC‐specific deletion of 5‐Ht2AR, further highlighting the critical role of the serotonin pathway in CRC.^115^

From the perspective of immunomodulation, Wang X et al. revealed that serotonin could potentiate anti‐tumour immunity via receptor‐independent mechanisms. Specifically, facilitated by TPH1 and SERT, CD8^+^ T cells achieve intracellular serotonin accumulation and subsequent serotonylation, which robustly induces anti‐tumour responses. Subsequent in vivo experiments further confirmed that serotonin significantly decreases tumour volume in murine MC38 subcutaneous models by activating the TPH1‐CAR‐T cell axis, thus highlighting the promising potential of serotonin in CRC immunotherapy.^116^

### 4.3 Therapeutic intervention strategies targeting Serotonin receptors in CRC

Given the critical regulatory role of serotonin in CRC oncogenesis and progression, modulating the synthesis, release, or receptor‐mediated signal transduction of peripheral serotonin has emerged as a promising intervention strategy to impede CRC progression. In recent years, the therapeutic efficacy of various serotonin receptor‐specific inhibitors in CRC has been progressively elucidated. By analysing clinical specimens, Sui H et al. reported that the protein expression levels of 5‐HT1DR, 5‐HT3CR, and 5‐HT4R are significantly greater in CRC than in normal colonic mucosa. Furthermore, compared with patients with low 5‐HT1DR expression, patients with high 5‐HT1DR expression have significantly shorter overall survival (OS) and disease‐free survival (DFS). In addition, a cohort study based on a Chinese population demonstrated that the long‐term, regular administration of selective serotonin reuptake inhibitors (SSRIs) is significantly negatively correlated with CRC incidence. This suppressive effect is particularly pronounced in advanced CRC, suggesting that SSRIs could serve as potent candidates for chemoprevention in high‐risk CRC patients.^117^

In vivo animal studies have revealed that the 5‐HT1DR inhibitor GR127935 can target Axin1 to suppress tumour metastasis in murine CRC models; moreover, in intestinal epithelial cells, serotonin binds to 5‐HT1DR to activate the Axin1/β‐catenin/matrix metalloproteinase‐7 (MMP‐7) signalling cascade, thus driving CRC invasion and migration. These mechanistic insights provide a robust experimental foundation for the clinical translation of 5‐HT1DR inhibitors.^118^ Similarly, Jun JH et al. reported that bisphenol A (BPA) exacerbates tumour burden in murine CRC subcutaneous models and significantly upregulates the expression of 5‐HT3 receptors in HT‐29 cells, indicating that the 5‐HT3 receptor may constitute a latent target mediating the carcinogenic effects of BPA.^119^ Furthermore, in vivo investigations have indicated that SSRIs can potentiate immunotherapeutic efficacy. In murine MC38 subcutaneous models, combined therapy using SSRIs and anti‐PD‐1 blockade yielded remarkable synergistic efficacy and increased CD8^+^ T cell functionality. These findings suggest that SSRIs may augment CD8^+^ T cell‐mediated anti‐tumour immunity by modulating intratumoral serotonin levels, thus potentiating the therapeutic effects of anti‐PD‐1 blockade.^120^ Clinically, a case‒control study revealed that the SSRI paroxetine, which is commonly prescribed for depression, can incrementally reduce the incidence of early‐onset colorectal cancer, further validating the tumour‐suppressive potential of SSRIs.^121^

**FIGURE 3:**
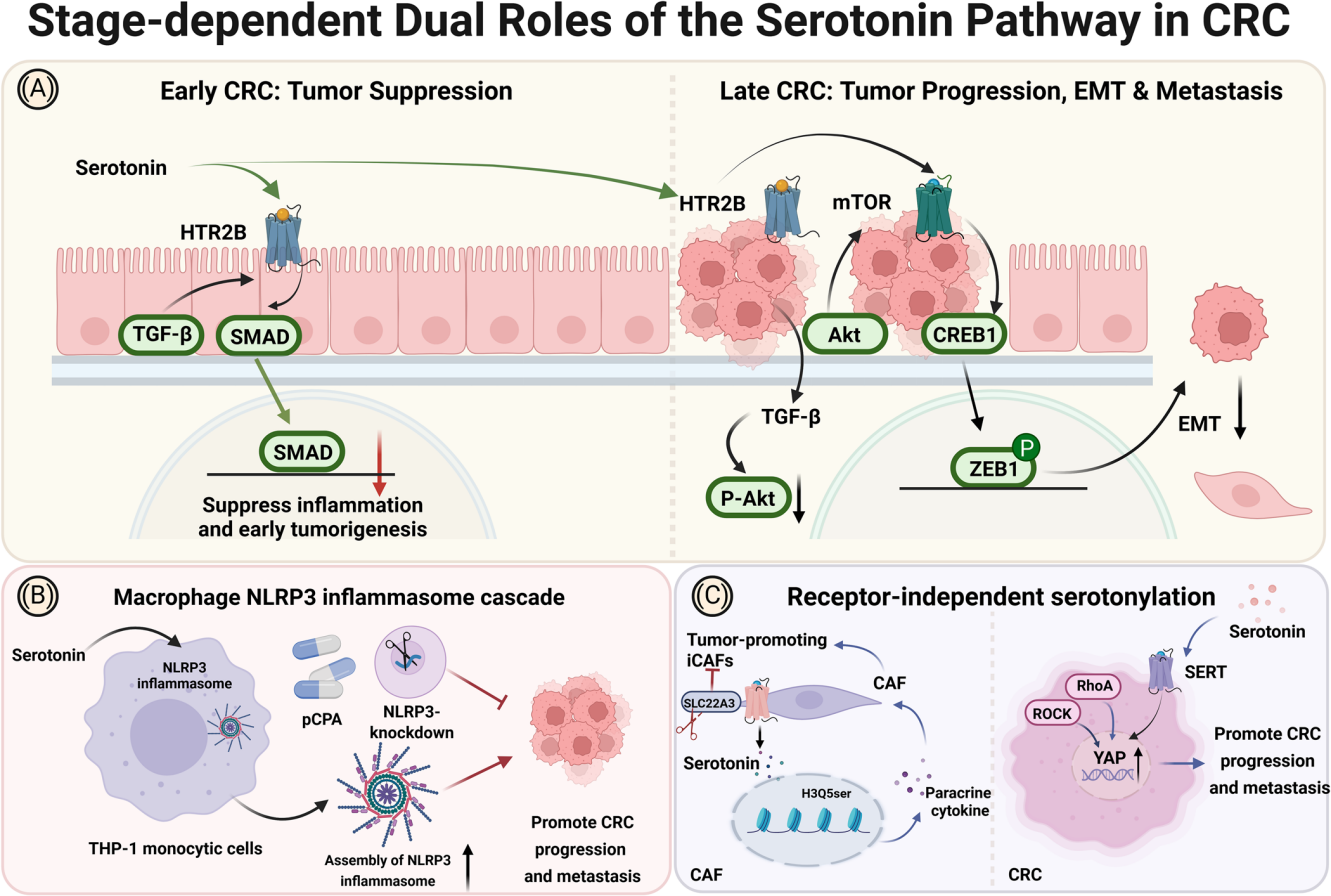
Stage‐dependent dual roles of the serotonin pathway in CRC. Dual functions of serotonin in early‐ and late‐stage CAC. In early lesions, serotonin‐HTR2B activates canonical TGF‐β/SMAD signalling to suppress inflammation and early tumorigenesis. In late‐stage CAC, serotonin‐HTR2B induces Akt phosphorylation (p‐Akt) through SMAD‐independent TGF‐β signalling to promote tumour proliferation and migration, and it can also induce EMT and metastasis through the CREB1‐ZEB1 pathway. (B) Macrophage NLRP3 inflammasome cascade. Serotonin promotes the assembly and activation of the NLRP3 inflammasome in THP‐1‐derived macrophages to drive colorectal cancer progression and metastasis. NLRP3 knockout or pCPA treatment can inhibit CRC growth and metastasis. (C) Receptor‐independent serotonylation mediates colorectal cancer progression. Serotonin can form covalent bonds with target proteins to produce receptor‐independent serotonylation modification. In CAFs, TGM2‐mediated H3Q5ser induces the iCAF phenotype and enhances malignant tumour phenotypes. SLC22A3 knockout or TGM2 inhibition can reverse CAF reprogramming. In CRC cells, SERT‐transported serotonin undergoes TGM2‐mediated RhoA serotonylation and activates the RhoA‐ROCK‐YAP pathway to promote tumour progression.

In summary, the serotonin metabolic pathway plays a dual role in CRC, which is orchestrated by receptor‐dependent and receptor‐independent mechanisms, dynamic signalling cascade transitions, and spatial modulation of the immune microenvironment. Collectively, the functional mechanisms of serotonin‐pathway‐related markers and the stage‐dependent regulatory network of this pathway in colorectal cancer are summarized in Table 2 and Figure 3.

**TABLE 2:** Functional Mechanisms of serotonin pathway‐related markers in CRC.

Molecule	Subject/Sample	Functional Mechanism	Reference
Serotonin	CRC patients /Mouse CRC model	Serotonin binds to HTR1B/1D/1F receptors to activate Wnt/β‐catenin signalling, thereby inhibiting colorectal CSC self‐renewal.	PMID: 35550066
Serotonin	Mouse CRC model	Serotonin enhances NLRP3 inflammasome activation; TPH1 inhibition or NLRP3 knockout suppresses tumour growth and metastasis.	PMID: 34285037
Serotonin	CRC cells	HTR2B activates the Akt/mTOR pathway to induce CREB1 phosphorylation, driving EMT, migration, and invasion.	PMID: 38381131
Serotonin	CRC cells	Inhibition of HTR2B blocks sustained ERK activation, suppressing CRC cell proliferation and migration.	PMID: 39255737
Serotonin	CRC patients/Serum	Elevated serum serotonin levels help distinguish CRC patients from healthy controls and assess prognosis.	PMID: 37046308
Serotonin	CRC cells	Serotonin promotes CRC progression by upregulating YAP expression via TMG2‐mediated serotonylation of RhoA.	PMID: 37046308
Serotonin	5‐Ht_2_AR	EGCs secrete CXCL10 and IL‐18: CXCL10 drives tumour‐infiltrating CD8^+^T cell recruitment, and IL‐18 potentiates CD8^+^T cell cytotoxicity. Consistently, EGC‐specific ablation of 5‐HT_2_AR fully abrogates the antitumour activity.	PMID: 42551424
Serotonin	CRC cells/Mouse CRC model	CD8^+^T cells undergo intracellular serotonin accumulation and serotonylation mediated by TPH1 and SERT, thereby inducing antitumour responses. In vivo studies demonstrate that serotonin activates the TPH1‐CAR‐T cell axis to shrink tumour volume in mice bearing MC38 subcutaneous tumours.	PMID: 38215751
Serotonin	CRC cells/Mouse CRC model	Serotonin exhibits a dual role, it suppresses tumour initiation via SMAD‐dependent TGF‐βsignaling but promotes proliferation in late stages via Akt.	PMID: 35664068
Serotonin	SSRI + PD‐1 antibody	Combined SSRI and anti‐PD‐1 therapy inhibits serotonin secretion by CD8^+^T cells, improving immunotherapeutic efficacy.	PMID: 40403728
SSRIs	CRC patients	Long‐term regular use of SSRIs reduces CRC risk, with more pronounced protective effects in advanced CRC.	PMID: 36497383
5‐HT1DR	5‐HT1DR inhibitor (GR127935)	Inhibition of 5‐HT1DR targets Axin1 to suppress CRC metastasis.	PMID: 26214021
5‐HTR2B	5‐HTR2B inhibitor (RS127445)	Inhibition of 5‐HTR2B reverses EMT and suppresses CRC metastasis.	PMID: 38381131

Abbreviations: Akt, protein kinase B; Axin1, axis inhibition protein 1; CRC, colorectal cancer; CXCL10, C‐X‐C motif chemokine ligand 10; EGC, enteric glial cells; EMT, epithelial‐mesenchymal transition; ERK, extracellular signal‐regulated kinase; IL‐18, interleukin‐18; mTOR, mammalian target of rapamycin; RhoA, ras homolog family member A; SMAD, mothers against decapentaplegic homolog; TGF‐β, transforming growth factor‐β; TGM2, transglutaminase 2; TPH1, tryptophan hydroxylase 1.

## 5 FUNCTIONAL ROLES OF THE INDOLE PATHWAY IN CRC

Within the tryptophan metabolic network, the kynurenine and serotonin pathways are governed primarily by host endogenous metabolism, whereas the indole pathway represents a predominantly gut microbiota‐dependent metabolic route. Advances in metabolomics and microbiomics have progressively elucidated the regulatory roles of indole derivatives in the pathogenesis, progression and therapeutic response of CRC. The underlying mechanisms involve the modulation of immune homeostasis, alterations in ferroptosis, and remodelling of the inflammatory microenvironment. A diverse array of indole derivatives can serve as ligands for AhR to facilitate its nuclear translocation, subsequently modulating the progression of colorectal cancer.^122^ Notably, the functions of indole metabolites in CRC are not uniform; instead, they play dualistic roles contingent upon specific metabolite profiles and concentration gradients. While certain metabolites possess pro‐tumorigenic activity, the majority exerts tumour‐suppressive effects, and a select few demonstrate bidirectional properties dictated by concentration thresholds or the local microenvironment.

### 5.1 Tumour‐suppressive mechanisms of indole metabolites

The majority of indole metabolites, including IAA, ILA and IPA, exhibit potent tumour‐suppressive activities, which are mediated through diverse mechanisms encompassing the regulation of intestinal stem cell homeostasis, the amelioration of the immune microenvironment and metabolic reprogramming.

The tumour‐suppressive effects of IAA are manifested primarily through the regulation of intestinal stem cells (ISCs) and the amelioration of inflammation. By investigating crypt‐resident microbiota within organoid models, Zhang S et al. demonstrated that IAA secreted by *Acinetobacter radioresistens* activates the AhR‐Wnt‐β‐catenin signalling axis to suppress ISC dedifferentiation and intestinal tumorigenesis in *Apc* ^Min/+^ mice, thus revealing the protective role of crypt‐resident microbiota during CRC pathogenesis.^123^ Concurrently, Wang J et al. confirmed that *Lactobacillus reuteri*‐derived IAA induces macrophages, T cells, and B cells to secrete IL‐35, which ameliorates the precancerous inflammatory microenvironment, alleviates colitis symptoms, and inhibits AOM/DSS‐induced CAC development.^124^ Furthermore, IAA directly suppresses CRC cell proliferation by activating Toll‐like receptor 4 (TLR4) and c‐Jun N‐terminal kinase (JNK) signalling.^125^ Furthermore, IAA targets *RNF32* to regulate colorectal cancer liver metastasis. In vitro siRNA‐mediated knockdown of *RNF32* suppresses the K48‐linked ubiquitination and degradation of GSK3β, upregulating GSK3β to block the Wnt/beta‐catenin signalling pathway. This cascade diminishes SPP1^+^ macrophage recruitment and inhibits tumour cell proliferation as well as epithelial–mesenchymal transition, thereby suppressing colorectal cancer progression.^126^

As the most extensively investigated tumour‐suppressive indole metabolite, ILA exhibits both AhR‐dependent barrier‐protective properties and dual AhR‐independent regulatory effects on metabolism and immunity. Consequently, it has been proven to play a pivotal role in restoring intestinal barrier integrity, facilitating metabolic reprogramming, and driving anti‐tumour immune activation. Research by Wang A et al. demonstrated that ILA derived from *Lactiplantibacillus plantarum* DPUL‐S164 serves as an AhR ligand to upregulate the nuclear factor erythroid 2‐related factor 2 (Nrf2) pathway while suppressing the NF‐κB cascade, thus promoting tight junction protein expression and ameliorating intestinal epithelial barrier damage.^127^ Furthermore, ILA can influence CRC progression via AhR‐independent pathways. Zhou S et al. reported that ILA directly binds to the SH2 domain of signal transducer and activator of transcription 3 (STAT3) and inhibits its phosphorylation at Tyr705. This abrogates the enrichment of p‐STAT3 at the hexokinase 2 (HK2) promoter region, downregulates the glycolytic cascade, and suppresses the Warburg effect in tumour cells to exert a robust tumour‐suppressive effect. Notably, the AhR antagonist CH223191 fails to attenuate the inhibitory effect of ILA on tumour cell viability, indicating that ILA operates primarily by directing metabolic reprogramming rather than directly modulating AhR. Clinical specimen analysis revealed that the expression levels of HK2 and p‐STAT3 are significantly elevated in CRC tissues and are negatively correlated with the abundance of ILA, further substantiating the metabolic regulatory function of ILA.^128^ Similarly, ILA has been shown to mediate the CRC risk‐reducing effects of statins via AhR‐independent pathways. Through retrospective cohort analyses and *Apc* ^Min/+^ murine model studies, Han JX et al. reported that atorvastatin significantly increased the abundance of *Lactobacillus reuteri*. Mechanistic investigations revealed that ILA produced by this bacterium targets the retinoic acid‐related orphan receptor γt (RORγt), which sequentially suppresses T helper 17 (Th17) cell differentiation and the activation of the interleukin‐17 (IL‐17) signalling cascade, thus slowing CRC progression.^129^ This study not only elucidates the anti‐cancer mechanism of statins but also highlights the chemopreventive potential of *Lactobacillus reuteri* and ILA against CRC.

In addition, Sugimura N et al. reported that ILA is a crucial effector molecule that mediates the anti‐tumour effects of *Lactobacillus gallinarum*. Their findings demonstrated that the abundance of *Lactobacillus gallinarum* is significantly diminished in the intestinal tracts of CRC patients; further in vivo and in vitro experiments confirmed that interventions with *Lactobacillus gallinarum* significantly reduce both the multiplicity and volume of intestinal tumours in murine models while concurrently modulating the structural composition of the gut microbiota. Furthermore, substantial enrichment of ILA was detected in both the conditioned medium of *Lactobacillus gallinarum* and the faeces of mice receiving this intervention.^122^ These findings confirm that ILA is the primary metabolite orchestrating the anti‐tumour efficacy of the strain, further underscoring the translational potential of ILA in CRC chemoprevention.

Dietary interventions can increase indole and ILA levels by reshaping the gut microbiota, thus profoundly influencing intestinal barrier integrity and tumour progression. The black rice diet (BRD) activates AhR and upregulates the transcription of AhR and its downstream target genes. This enhances tryptophan metabolism, thus increasing the levels of L‐tryptophan, indole, and ILA, which collectively suppress CRC cell proliferation and colorectal tumorigenesis. These three metabolites are positively correlated with the abundance of *Lactobacillus johnsonii*, demonstrating that BRD impedes intestinal tumour progression by fostering beneficial effects on the gut microbiota and its associated metabolites.^130^ Clinical investigations have revealed that the expression of the barrier protein ZO‐1 in CRC patients is positively correlated with the indole/TRP ratio. Moreover, the abundances of *Parabacteroides* and Firmicutes are significantly decreased in the faeces of CRC patients, and the prevalence of these microbial taxa is positively correlated with faecal indole levels.^70^ These findings suggest that microbiota‐mediated alterations in tryptophan metabolism may compromise intestinal barrier function and contribute to CRC pathogenesis, thus offering novel therapeutic paradigms for CRC management.

ILA also drives epigenetic remodelling to orchestrate the anti‐tumour immune microenvironment. Specifically, ILA produced by *Lactobacillus plantarum* L168 enhances anti‐tumour immunity via epigenetic modulation: by upregulating H3K27ac enrichment at the enhancer region of *IL12a*, ILA promotes IL‐12a secretion by dendritic cells, which subsequently triggers CD8^+^ T cell‐mediated anti‐tumour immunity. Concurrently, ILA increases chromatin accessibility to downregulate the expression of the cholesterol metabolism‐associated gene *Saa3* in CD8^+^ T cells, thus promoting the functionality of tumour‐infiltrating CD8^+^ T cells. These epigenetic mechanisms strongly underscore the therapeutic potential of ILA in CRC.^131^

In addition to ILA and IAA, IPA and ICA equally possess robust tumour‐suppressive activities. IPA and ICA produced by *Limosilactobacillus fermentum* GR‐3 effectively suppress AOM/DSS‐induced tumorigenesis by modulating the gut microbiota and mitigating oxidative stress and inflammation.^132^

Xu H et al. engineered the bacterial construct LR‐S‐CD/CpG@LNP; this system uses reactive oxygen species (ROS)‐responsive linkers to conjugate an immune adjuvant (CpG) and carbon dot (CD) co‐loaded plant lipid nanoparticles (CD/CpG@LNPs) onto the surface of *Limosilactobacillus reuteri* (*LR)*. This construct significantly enhances the tumoral infiltration of cytotoxic T lymphocytes, thus suppressing CRC. Simultaneously, LR alters tryptophan metabolism to upregulate I3A levels, which robustly amplifies the anti‐tumour immune response. This dual mechanism strongly suggests that indole metabolites can serve as efficacious sensitizers for immunotherapy.^133^

Notably, while earlier studies predominantly focused on the tumour‐suppressive effects of indole derivatives synthesized by single bacterial strains, recent paradigm shifts revealed that the gut microbiota could synergistically generate indole metabolites with diverse biological activities to collectively influence tumour cell functionality. For example, the intestinal‐resident *Lactobacillus johnsonii* can cooperatively metabolize TRP alongside *Clostridium sporogenes* to biosynthesize IPA. IPA significantly enhances the sensitivity of multiple malignancies‐including melanoma, breast cancer, and CRC to anti‐PD‐1 immunotherapy.^134^ These findings underscore that investigations into the impact of the indole metabolic pathway on CRC should not be restricted to isolated single strains; instead, the profound implications of synergistic microbial metabolism must be comprehensively integrated. Considering that intestinal microecology fundamentally operates as a holistic continuum, this multi‐strain collaborative research approach more accurately reflects the in situ pathological dynamics during tumorigenesis.

### Procarcinogenic mechanisms of indole derivatives

In addition, a subset of indole metabolites, including 3‐methylindole, IDA and IS, has been shown to facilitate CRC pathogenesis. Indole metabolites synthesized by certain pathogenic bacteria similarly possess AhR ligand activity; by inducing hyperactivation of AhR, these compounds remodel the tumour microenvironment and inhibit ferroptosis, thus exerting potent pro‐tumorigenic effects.

Ishii K et al. reported that 3‐methylindole triggers inflammatory responses in Caco‐2 cells by activating the extracellular signal‐regulated kinase (ERK) and p38 MAPK pathways, which subsequently drive NF‐κB activation and upregulate the expression of pro‐inflammatory cytokines such as IL‐6 and TNF‐α. Furthermore, AhR activation partially antagonizes NF‐κB activity and the release of inflammatory mediators, suggesting that 3‐methylindole modulates inflammation through the homeostatic balance between NF‐κB and AhR signalling, thus contributing to the pathogenesis of CRC and inflammatory bowel disease (IBD).^135^ Kurata K et al. further demonstrated that 3‐methylindole activates the synergistic coupling of p38 and c‐Jun N‐terminal kinase (JNK) to upregulate TNF‐α expression in IEC; this elevated TNF‐α subsequently drives IEC apoptosis via autocrine and paracrine signalling, an effect that can be partially mitigated by AhR activation.^136^ Moreover, clinical evidence has indicated that 3‐methylindole levels are significantly elevated in the faeces and breath of CRC patients, highlighting its potential as a volatile biomarker for CRC screening.^137^

Furthermore, the pro‐tumorigenic role of IDA has been substantiated. Research has indicated that IDA, a metabolite derived from *Peptostreptococcus anaerobius*, acts as an endogenous ligand for AhR; its activation of AhR upregulates the expression of aldehyde dehydrogenase 1 family member A3 (*ALDH1A3*), which utilizes retinaldehyde to generate NADH. This process inhibits ferroptosis suppressor protein 1 (FSP1)‐mediated synthesis of reduced coenzyme Q10, thus suppressing tumour cell ferroptosis and promoting CRC development. Further investigations revealed a significant enrichment of *Peptostreptococcus anaerobius* in CRC patients; notably, both IDA and *Peptostreptococcus anaerobius* accelerated CRC progression in murine models, providing an experimental rationale for targeting the IDA‐AhR‐ALDH1A3 axis in CRC therapy.^8^ These findings suggest that the regulation of AhR by indole metabolites is strictly ligand dependent; consequently, when prebiotics and intervention strategies targeting indole metabolism are designed, circumventing the pro‐tumorigenic effects driven by sustained AhR hyperactivation is imperative.

As a uremic toxin, IS promotes the proliferation of HCT‐116 cells by activating the Akt/β‐catenin/c‐Myc and AhR/c‐Myc signalling cascades; furthermore, the Akt inhibitor MK2206, the AhR antagonist CH223191, and the c‐Myc inhibitor 10058‐F4 attenuate the IS‐induced increase in EGFR protein expression. These findings indicate that IS enhances EGF signalling, thus increasing the risk of CRC in patients with chronic kidney disease.^138^

Venkateswaran N et al. reported that *Fusobacterium nucleatum*‐derived IPA activates AhR in macrophages, driving M2 polarization to establish an immunosuppressive tumour microenvironment that promotes the malignant progression of CRC.^139^ Similarly, cooperative microbial metabolism actively participates in the generation of pro‐tumorigenic metabolites. Research has shown that the concentration of IPYA is markedly elevated in the co‐culture supernatant of the archaeon *Methanobrevibacter smithii* ALI and *Fusobacterium nucleatum*.^140^ Furthermore, in vitro and in vivo experiments have demonstrated that exogenous IPYA supplementation promotes MYC‐driven hepatocellular carcinoma, suggesting that IPYA serves as a critical regulatory metabolite that drives tumour growth.^141^ Currently, direct investigations regarding the role of IPYA in CRC are lacking, and its precise function in CRC pathogenesis warrants further validation through animal models and clinical cohorts.

### 5.2 Concentration‐dependent dual role of IAAD in colorectal cancer

While current studies predominantly classify indole metabolites into tumour‐suppressive and pro‐tumorigenic categories on the basis of biological outcomes, the regulatory functions of certain indole derivatives are not static but exhibit pronounced concentration dependency, with IAAD serving as a prime example. Dai Z et al. reported that IAAD at low concentrations induces HCT116 apoptosis, suppresses cellular proliferation, and impedes tumour progression in murine CRC models; conversely, at high concentrations, IAAD promotes the epithelial‒mesenchymal transition (EMT), invasion, and metastasis in CRC cells by activating AhR and binding to target proteins such as matrix metalloproteinase‐9 (MMP9) and angiotensin‐converting enzyme (ACE).^142^ Notably, while IAAD upregulated the transcription of the AhR target genes *CYP1A1* and *CYP1B1* and induced apoptosis in HCT116 cells, it did not significantly affect the apoptosis rate of DLD‐1 cells. Further mechanistic investigations revealed that the baseline AhR activation level is significantly greater in HCT116 cells than in DLD‐1 cells, indicating that intercellular variations in AhR expression and activation capacity dictate differential cellular responsiveness to IAAD.

Furthermore, emerging evidence indicates that the traditional Chinese medicine Mangxiao suppresses CRC by ameliorating tryptophan metabolism disorders, with IAAD identified as one of the significantly altered differentially abundant metabolites following Mangxiao intervention. These findings suggest that IAAD might be a promising therapeutic target for integrative traditional Chinese and Western medicine in CRC management.^143^ The regulatory mechanisms underlying the bidirectional role of IAAD are hypothesized to be linked to the activation intensity of specific signalling pathways by the metabolite; however, the precise molecular mechanisms, concentration thresholds, and actual in vivo effects within the tumour microenvironment warrant further investigation.

In summary, the concentration‐dependent, bidirectional role of IAAD highlights the functional complexity of indole metabolites in CRC, precluding any simplistic generalization as strictly tumour‐suppressive or pro‐tumorigenic. This dose‐dependent characteristic provides a novel rationale for targeted interventions in CRC while offering fresh perspectives for understanding the functional diversity of indole metabolites.

### 5.3 Limitations and future research directions of microbiota‒indole‒host interactions

Notably, current investigations concerning indole metabolites and CRC predominantly rely on correlation analyses linking microbial abundance, metabolite levels, and tumour phenotypes. Lacking robust experimental validation bridging correlation and causality, the overarching “microbiota–metabolite–host” causal axis remains incomplete. To overcome the limitations of existing studies, which largely illustrate unidirectional regulatory relationships among the microbiota, indole metabolites, and CRC and to precisely elucidate the specific mechanisms of action of indole metabolites, future research must establish a comprehensive causal validation framework. First, the causal link between specific microbial consortia and tumour phenotypes should be established utilizing germ‐free animal colonization or faecal microbiota transplantation (FMT). Second, the independent effects of indole derivatives should be validated by employing metabolic enzyme knockout or complementation strains alongside wild‐type controls. Finally, downstream molecular targets should be confirmed by integrating host receptor knockout or pathway inhibition assays.

Nevertheless, each of these conventional experimental modalities has inherent limitations. First, germ‐free animals exhibit developmental defects in their immune systems, rendering them incapable of fully replicating the complex tumour immune microenvironment of CRC under physiological conditions. Second, FMT reflects only macroscopic microbial effects and is constrained by donor microbiota heterogeneity and recipient colonization efficiency, making it difficult to precisely pinpoint core bacterial strains or key metabolites. Third, most gut anaerobes lack mature genetic editing systems, presenting significant hurdles for genetic manipulation, while their intricate metabolic networks readily trigger bypass compensatory effects that obscure the true phenotypes of single‐pathway knockouts.

To overcome these technical bottlenecks, future research can integrate diverse experimental methods to achieve synergistic complementarity. With respect to microbial interventions, standardized synthetic microbial communities can replace complex FMT to identify pivotal active strains. Furthermore, inhibitors targeting bacterial metabolic enzymes can be developed to bypass the technical hurdles of anaerobic gene editing, and co‐culture models comprising human immune cell‐derived organoids and bacterial strains can be established to compensate for the immunological deficiencies of germ‐free mice. Metabolically and clinically, stable isotope tracing can be integrated with clinical mediation analyses that adjust for confounding variables such as age, diet, and chronic inflammation. Combined with metagenomic and metabolomic profiling to dynamically track fluctuations in TRP substrates and microbial metabolites within the tumour microenvironment, this multi‐faceted approach will more accurately and comprehensively elucidate the mechanisms of indole metabolites in CRC, providing a robust theoretical foundation for subsequent clinical translation.

### 5.4 Translational value and immunotherapy‐sensitizing potential of indole derivatives

In recent years, the translational potential of gut microbiota‐derived indole metabolites as immunotherapeutic adjuvants has garnered substantial interest, as these compounds have demonstrated remarkable efficacy in enhancing CRC immunotherapy outcomes. The primary limitation in current CRC immunotherapy is the low response rates of the majority of MSS CRC patients to immune checkpoint inhibitors. The gut microbiota and its derived indole derivatives provide novel intervention targets to overcome this barrier.

As a pivotal product of tryptophan metabolism by the gut microbiota, IPA exhibits prominent sensitizing effects in CRC immunotherapy. Jia et al. reported that IPA upregulates H3K27ac modification levels, activates the expression of the *Tcf7* super‐enhancer, and promotes TCF‐1 expression, thus expanding functional progenitor‐derived CD8^+^ T cell populations. This robustly potentiates anti‐PD‐1 immunotherapeutic efficacy across multiple tumour models, including melanoma, breast cancer, and colorectal cancer, providing direct empirical evidence that IPA is a viable CRC immunotherapy adjuvant.^134^

Furthermore, ICA derived from *Lactobacillus gallinarum* has been confirmed to significantly increase anti‐PD‐1 immunotherapeutic efficacy in CRC. ICA plays multifaceted roles through the regulation of the IDO1/KYN/AhR signalling axis. Specifically, it suppresses IDO1 expression in tumour cells to reduce the production of the immunosuppressive metabolite KYN, and it competitively binds to AhR against KYN, thus blocking KYN‐induced CD4^+^ Treg differentiation. In vivo experiments demonstrated that ICA diminishes intratumoral Treg infiltration while promoting CD8^+^ effector T‐cell functions, significantly increasing anti‐PD‐1 efficacy in both MSS and dMMR murine CRC models. Moreover, exogenous KYN supplementation reverses this therapeutic effect, further confirming that the immunosensitizing activity of ICA relies heavily on the IDO1–KYN–AhR pathway. Notably, ICA remains equally efficacious against MSS‐type CRC, which typically has low responsiveness to immunotherapy, thus offering a novel avenue to overcome current therapeutic barriers in MSS CRC management.^144^

**FIGURE 4:**
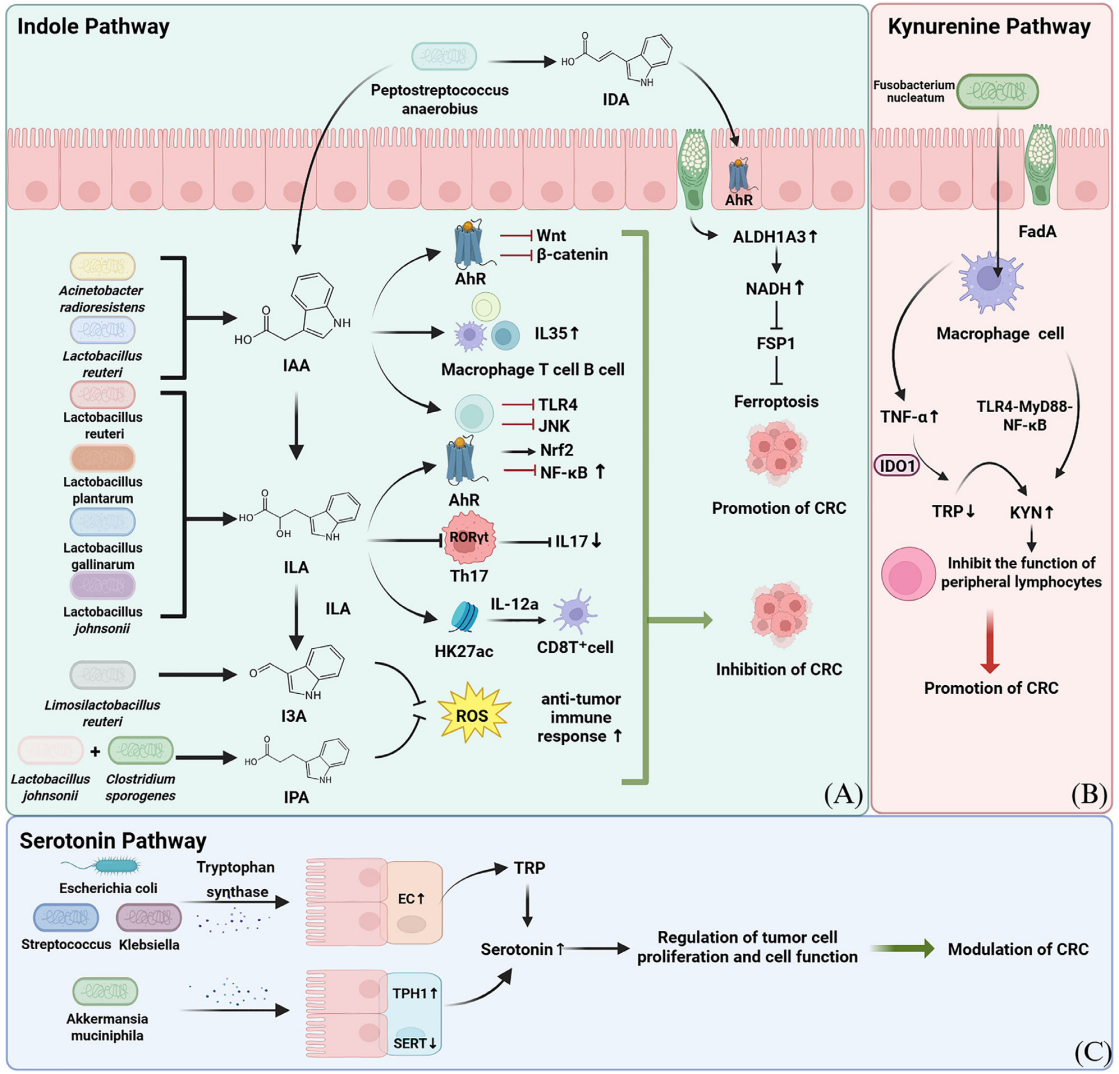
Proposed mechanistic model of gut‐microbiota‐derived tryptophan metabolites in CRC. (A) Indole pathway: This pathway exerts dual regulatory effects on CRC progression. Pro‐tumour: *Peptostreptococcus anaerobius* produces indoleacrylic acid (IDA), which activates the aryl hydrocarbon receptor (AhR) and upregulates ALDH1A3 to suppress tumour‐cell ferroptosis and promote CRC progression. Anti‐tumour: *Lactobacillus reuteri* and *Acinetobacter radioresistens* generate indole‐3‐acetic acid (IAA); Lactobacillus plantarum and other lactobacilli produce indole‐3‐lactic acid (ILA); *Limosilactobacillus reuteri* yields indole‐3‐carboxaldehyde (I3A); *Lactobacillus johnsonii* and *Clostridium sporogenes* produce indole‐3‐propionic acid (IPA). IAA acts via AhR‐Wnt‐β‐catenin signalling, represses the TLR4‐JNK pro‐inflammatory pathway, and promotes IL‐35 secretion by immune cells. ILA targets RORγt to inhibit Th17‐cell differentiation, activates AhR, upregulates nuclear factor erythroid 2‐related factor 2 (Nrf2) while inhibiting NF‐κB signalling, increases H3K27ac enrichment at the Il‐12a enhancer, and elicits CD8^+^ T cell‐mediated anti‐tumour immunity. I3A and IPA trigger reactive oxygen species (ROS) accumulation to enhance anti‐tumour immune responses and restrain CRC development. (B) Kynurenine pathway: *Fusobacterium nucleatum* invades macrophages through its surface adhesin FadA, activates TLR4‐MyD88‐NF‐κB signalling, and upregulates macrophage IDO1 expression via TNF‐α. This induces local tryptophan (TRP) depletion and robust kynurenine (KYN) accumulation, suppresses peripheral lymphocyte function and drives CRC progression. (C) Serotonin pathway: *Escherichia coli, Streptococcus, and Klebsiella* express tryptophan synthase to supply tryptophan precursors for serotonin biosynthesis. *Akkermansia muciniphila* upregulates tryptophan hydroxylase 1 (TPH1) and downregulates the serotonin transporter (SERT), modulating tumour cell proliferation and biological functions and contributing to CRC pathogenesis.

In summary, indole metabolites hold remarkable translational value as immunotherapeutic adjuvants. By augmenting CD8^+^ T cell‐mediated immunity, suppressing immunosuppressive Treg differentiation, and blocking the KYN‐AhR immunosuppressive cascade, these findings provide novel strategies to improve CRC immunotherapy outcomes. Future preclinical investigations should precisely delineate the optimal therapeutic concentrations, combination treatment strategies, and clinical translational pathways of diverse indole metabolites, followed by clinical trials to validate their effectiveness as immunological adjuvants. Collectively, the functional characteristics of indole‐derived metabolites and the integrated mechanistic model of gut‐microbiota‐originated tryptophan metabolites in colorectal cancer are presented in Table 3 and Figure 4.

**TABLE 3:** Functional mechanisms of indole pathway‐related markers in CRC.

Molecule	Subject/Sample	Functional Mechanism	Reference
IDA	Mouse CRC model/CRC patients	IDA activates AhR and inhibits tumour cell ferroptosis, thereby promoting CRC progression.	PMID: 38168770
ILA	CRC cells/Mouse CRC model	ILA enhances the anti‐tumour immune response of CD8^+^ T cells and downregulates the glycolytic pathway to inhibit the Warburg effect in tumour cells, thereby suppressing CRC progression.	PMID: 40409349
ILA	CRC cells/Mouse CRC model	ILA suppresses CRC progression by inhibiting Th17 cell differentiation and the activation of the IL‐17 signalling pathway.	PMID: 37069401
ILA	Mouse CRC model	A black rice diet activates AhR and enhances tryptophan metabolism, increasing the levels of L‐Trp, indoles, and ILA, thereby inhibiting CRC progression.	PMID: 38868519
IAA	CRC organoids	IAA inhibits the transition of ISCs, thereby preventing CRC initiation.	PMID: 39972061
IAA	Mouse CRC model	IAA induces IL‐35 secretion from macrophages, T cells, and B cells, improving the pre‐cancerous inflammatory microenvironment to inhibit CAC initiation.	PMID: 40274281
IAA	CRC cells/Mouse CRC model	IAA is an RNF32 inhibitor. In vitro, siRNA‐mediated RNF32 knockdown suppresses K48‐linked ubiquitin‐dependent degradation of GSK3β. Elevated GSK3β blocks Wnt/β‐catenin signalling, reduces SPP1^+^ macrophage recruitment, and inhibits tumour cell proliferation, EMT and CRC progression.	PMID: 41387204
ILA	CRC cells/Mouse CRC model	ILA activates CD8^+^ T cell‐mediated antitumour immunity and downregulates the cholesterol metabolism‐related gene *Saa3* to inhibit CRC.	PMID: 37192617
3‐Methylindole	CRC cells	It activates ERK/p38 MAPK and NF‐κB while also activating AhR to partially inhibit inflammatory cytokine release.	PMID: 39451248
3‐Methylindole	CRC patients/ Faecal, Breath	Elevated 3‐methylindole levels serve as diagnostic biomarkers in faecal and breath samples.	PMID: 37406626
IAAD	CRC cells/Mouse CRC model	IAAD induces apoptosis and delays CRC progression at low concentrations, but activates AhR and binds to MMP9 and ACE at high concentrations to promote CRC development.	PMID: 38994159
IAAD	Mouse CRC model	Mirabilite inhibits CRC by modulating tryptophan metabolism, with IAAD identified as a key differential metabolite significantly altered after intervention.	PMID: 35528071
IPA, ICA	Mouse CRC model	IPA and ICA modulate the gut microbiota, reduce oxidative stress and inflammation, and inhibit CRC initiation.	PMID: 39242568
IPA	Mouse CRC model	Intestinal *Lactobacillus johnsonii* and *Clostridium sporogenes* synergistically metabolize TRP to generate IPA. IPA elevates H3K27ac levels, activates the *Tcf7* super‐enhancer to promote TCF‐1 expression, and sensitizes CRC to anti‐PD‐1 immunotherapy.	PMID: 38490195
IPA	CRC cells/Mouse CRC model	Fusobacterium nucleatum‐derived IPA activates AhR in macrophages, triggers M2‐type polarization, shapes an immunosuppressive tumour microenvironment, and promotes malignant progression of CRC.	PMID: 40174685
ICA	CRC cells/Mouse CRC model	Lactobacillus gallinarum‐derived ICA modulates the IDO1‐KYN‐AhR axis to reduce KYN production. ICA competes with KYN for AhR binding, blocks KYN‐driven CD4^+^ Treg differentiation, and enhances anti‐PD‐1 therapeutic efficacy in CRCPMC	PMID: 37770127
I3A	Mouse CRC model	The engineered bacteria LR‐S‐CD/CpG@LNP modulate tryptophan metabolism, upregulating I3A levels and amplifying antitumour immune responses.	PMID: 39739630
IS	CRC cells	IS activates Akt/β‐catenin/c‐Myc and AhR/c‐Myc pathways and enhances EGF signalling, thereby increasing the risk of CRC in patients with chronic kidney disease.	PMID: 39852970

Abbreviations: ACE, angiotensin‐converting enzyme; AhR, aryl hydrocarbon receptor; Akt, protein kinase B; c‐Myc, cellular myelocytomatosis oncogene; CRC, colorectal cancer; EMT, epithelial‐mesenchymal transition; ERK, extracellular signal‐regulated kinase; GSK3β, glycogen synthase kinase 3β; H3K27ac, histone H3 lysine 27 acetylation; I3A, indole‐3‐aldehyde; IAA, indole‐3‐acetic acid; IAAD, indoleacrylic acid; ICA, indole‐3‐carboxylic acid; IDA, indole‐3‐acetic acid; IL‐35, interleukin‐35; ILA, indole‐3‐lactic acid; IPA, indole‐3‐propionic acid; IS, indoxyl sulphate; ISC, intestinal stem cell; L‐Trp, L‐tryptophan; MMP9, matrix metalloproteinase 9; PD‐1, programmed cell death protein 1; RNF32, RING finger protein 32; Saa3, serum amyloid A3; SPP1, secreted phosphoprotein 1; TCF‐1, T‐cell factor 1; Tcf7, transcription factor 7; Wnt, wingless‐related integration site; β‐catenin, beta‐catenin.

## 6 CONCLUSION AND FUTURE PERSPECTIVES

### 6.1 Conclusion

The three key pathways of tryptophan metabolism regulate CRC initiation and progression through diverse molecular mechanisms, offering novel insights into CRC pathogenesis and advancing clinical translation. Overall, tryptophan metabolism exerts complex, bidirectional regulatory effects in CRC. Substrate competition among the three pathways, pathway compensation, the dual functions of AhR, and the involvement of stromal cells such as CAFs likely mediate these dual effects, which also partially account for the suboptimal clinical efficacy of IDO‐targeted therapies.

Centred on the core rate‐limiting enzymes IDO1 and TDO, the KP represents the primary metabolic branch of tryptophan in humans. Moderate KP activation during early inflammation repairs the intestinal barrier and inhibits inflammation‐driven carcinogenesis, whereas sustained KP hyperactivation within the tumour microenvironment drives immunosuppression. The KYN/TRP ratio and IDO1 expression level hold potential as diagnostic and prognostic biomarkers for early‐stage CRC. Furthermore, IDO1 inhibitors combined with PD‐1 and HDAC inhibitors demonstrate synergistic anti‐tumour efficacy in MSS CRC. Downstream metabolites of the KP display functional heterogeneity: KYN mediates immune tolerance by activating AhR, whereas QA is associated with adverse CRC prognosis. In addition, certain gut bacteria can directly synthesize kynurenine‐like metabolites to modulate the tumour microenvironment.

Accounting for merely 1% to 2% of total tryptophan metabolism, the serotonin pathway similarly plays a dual role in CRC. Selective serotonin reuptake inhibitors (SSRIs) exhibit chemopreventive potential in high‐risk CRC patients, and their combination with PD‐1 inhibitors offers novel strategies for CRC immunotherapy. Furthermore, the gut microbiota participates in CRC progression by regulating serotonin synthesis and utilization, serving as a potential therapeutic target. This pathway exhibits pronounced stage‐specific characteristics, with 5‐HT2B displaying tumour‐suppressive properties during early‐stage CRC but mediating pro‐tumorigenic effects in advanced stages, whereas 5‐HT2AR expressed on EGC serves as a potential antitumour target. However, the functions of other receptor subtypes, such as 5‐HT1DR and 5‐HT3R, remain elusive, and most 5‐HTR inhibitors have been validated only in animal models; thus, phase II/III clinical trials in CRC patients are lacking. The molecular mechanisms governing the bidirectional role of serotonin across distinct tumour microenvironments also await elucidation.

The indole pathway is involved in gut microbiota‐dependent tryptophan metabolism and plays a pivotal role in microbiota–metabolite–host interactions during CRC. Indole metabolites derived from diverse microbial sources exhibit stark functional differences. *Lactobacillus*‐derived ILA and IPA repair the intestinal barrier and activate CD8^+^ T cells to exert anti‐tumour effects, whereas pathogen‐derived IDA and 3‐methylindole inhibit ferroptosis, exacerbate intestinal inflammation and promote tumour progression. IAAD displays concentration‐dependent bidirectional effects, inducing CRC apoptosis at low concentrations while promoting tumour EMT via AhR activation at high concentrations. Microbiota‐targeted interventions, such as engineered bacterial modifications and probiotic administration, can exert anti‐tumour effects by reshaping indole metabolic patterns, demonstrating favourable therapeutic potential.

From a clinical translational perspective, tryptophan metabolic dysregulation supports the use of metabolome‐ and microbiome‐stratified personalized therapy for CRC. Patients with tryptophan metabolism hyperactivation are more likely to benefit from IDO inhibitors combined with immunotherapy, whereas those with activated compensatory pathways exhibit limited responses to single‐targeting agents, necessitating multi‐pathway combinatorial interventions. However, the current lack of a standardized stratification system for tryptophan metabolism, compounded by the bidirectional regulatory properties of the metabolites themselves, limits the standardized clinical application of targeted therapies. Furthermore, the multi‐organ tryptophan metabolism network broadens the perspective of CRC mechanistic research. Tryptophan metabolites generated in extraintestinal tissues, such as the liver and immune organs, can mediate inter‐organ communication via the circulatory system, thus participating in CRC initiation and progression.

### 6.2 Future perspectives

Although progress has been made regarding tryptophan metabolism in CRC, unclear mechanisms and clinical translation hurdles persist, and require further investigation.

Research on the KP has focused predominantly on the rate‐limiting enzymes IDO1 and TDO, while the regulation and functions of downstream metabolic enzymes such as KMO and KYNU remain to be fully elucidated. Clinical translation of IDO1 inhibitors faces several hurdles, including lower‐than‐expected efficacy of monotherapies and combination regimens, alongside off‐target effects. Most studies lack direct pharmacodynamic validation of local tumour target engagement, and single‐target inhibitors fail to circumvent IL4I1‐mediated bypass resistance, making clinical efficacy difficult to reproduce. The diagnostic value of individual KP metabolites is limited, and the distinct functions of various metabolites, along with uncertainties surrounding microbiota‐induced KP metabolic reprogramming, warrant clinical validation regarding potential risks. Future efforts can focus on downstream metabolic enzymes, in situ tumour pharmacodynamic evaluation, and bypass resistance mechanisms to optimize combination treatment strategies. Integrating multi‐omics markers from peripheral blood and stool samples will also help establish monitoring models for high‐risk populations with IBD or familial CRC, alongside the development of IDO1/IL4I1 dual‐target inhibitors and the advancement of selective AhR antagonists and KMO inhibitors.

The serotonin pathway suffers from poorly defined receptor subtype functions and a paucity of clinical evidence. Subsequent studies should prioritize dissecting the stage‐specific functions of each receptor subtype and promoting clinical trials of SSRIs and related inhibitors to evaluate their translational value.

Several deficiencies of the indole pathway have been identified, including unclear target proteins, unknown synergistic or antagonistic effects among various metabolites and microbiota, a lack of systematic validation of the microbiota–metabolite–host regulatory network, and unelucidated molecular mechanisms underlying the concentration‐dependent effects of certain metabolites. Subsequent studies must clarify the modes of action between indole metabolites and target molecules and dissect the molecular mechanisms governing the combined effects of multiple metabolites. Leveraging microbial and dietary interventions such as engineered bacterial modifications, probiotic and prebiotic screening, and FMT can improve the tumour metabolic and immune microenvironment, thus achieving stratified personalized therapy for CRC.

**FIGURE 5:**
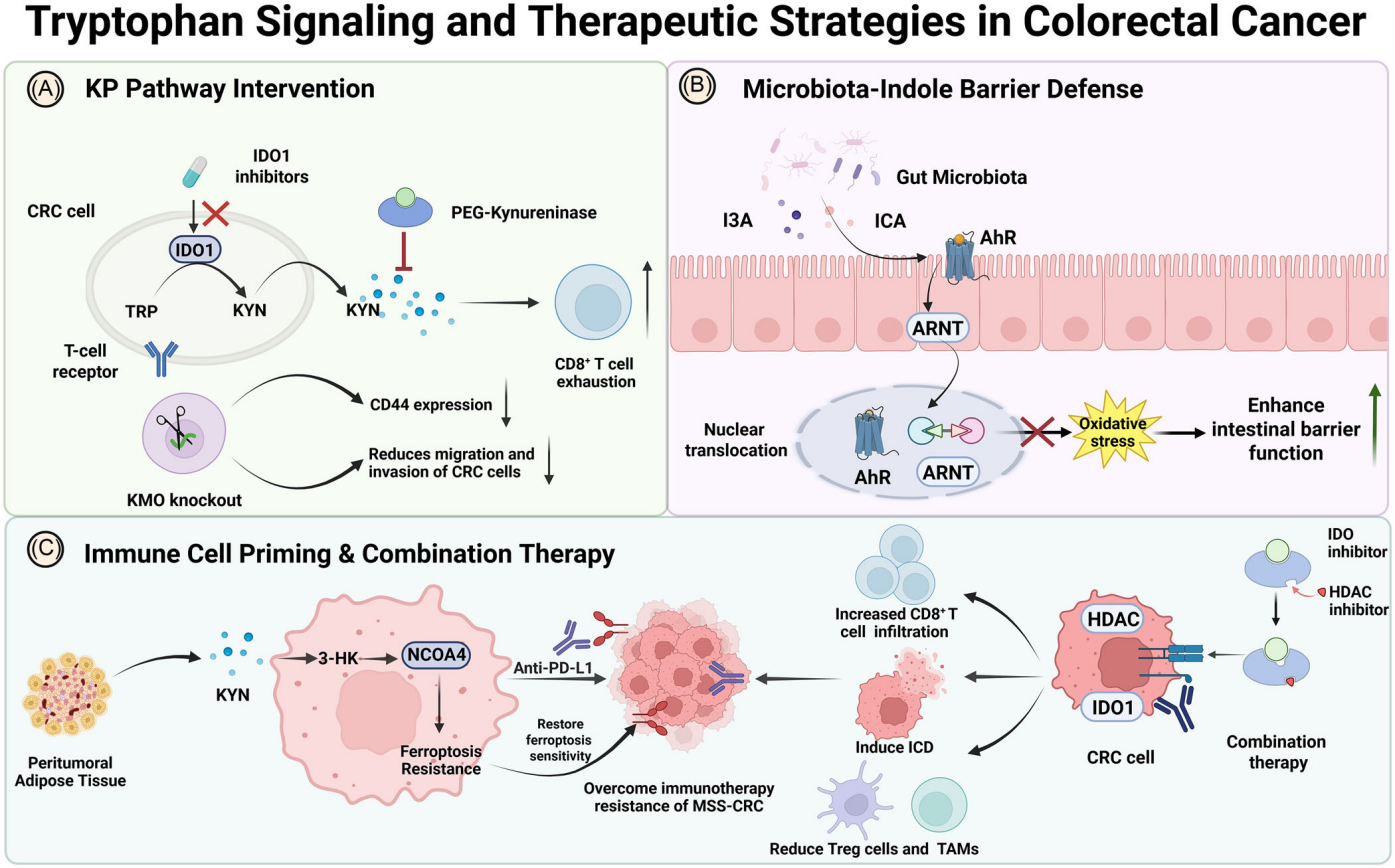
Tryptophan signalling and therapeutic strategies in colorectal cancer. (A) KP pathway intervention. IDO1 inhibitors block IDO1‐catalysed conversion of tryptophan (TRP) to kynurenine (KYN). PEG‐kynureninase degrades KYN to alleviate CD8^+^ T‐cell exhaustion. KMO knockout downregulates CD44 expression and inhibits migration and invasion of colorectal cancer cells. (B) Microbiota‐indole‐mediated intestinal barrier defence. Gut‐microbiota‐derived indole metabolites I3A and ICA activate the AhR‐ARNT signalling complex. Nuclear translocation of AhR‐ARNT suppresses oxidative stress and enhances intestinal barrier function. (C) Immune‐cell priming and combination therapy. Peritumoral adipose tissue‐derived kynurenine is metabolized by tumour cells into 3‐HK, which binds to NCOA4 and induces ferroptosis resistance. Inhibition of the kynurenine pathway improves anti‐PD‐1 therapeutic efficacy and overcomes immunotherapy resistance in MSS‐CRC. Combined IDO and HDAC inhibitor treatment induces immunogenic cell death (ICD), increases CD8^+^ T‐cell infiltration, reduces Treg and tumour‐associated macrophage (TAM) populations, and reverses immunosuppressive effects.

Overall, a substantial body of evidence stems from cellular and animal experiments, while causal relationships in the microbiota–metabolite–host axis and tryptophan cross‐organ metabolic communication still lack clinical cohort support. Furthermore, substrate competition, stromal cell regulatory effects, and effective concentration thresholds of metabolites require further investigation. Future research can integrate metagenomics, metabolomics, stable isotope tracing, and human organoid models to conduct large‐scale prospective clinical studies, thus establishing a comprehensive pathway for translating basic research on tryptophan metabolism into clinical prevention and treatment for CRC. Multiple therapeutic interventions targeting tryptophan‐related signaling and combinatorial treatment options for colorectal cancer are summarized in Figure 5.

## AUTHOR CONTRIBUTIONS

Study concept and design: Yuepeng Cao and Yuping Zhou. Literature review and original drafting of the manuscript: Ran Wang. Collecting references: Ran Wang, Wenke Shen and Dongxue Yang. Figure and table preparation: Wenke Shen and Dongxue Yang. Writing review and editing: Ping Yang, Yuepeng Cao and Yuping Zhou. Funding acquisition: Ping Yang, Yuping Zhou and Dongxue Yang. All the authors have reviewed and approved the final version of the manuscript.

## CONFLICT OF INTEREST STATEMENT

The authors declare no conflicts of interest.

## ETHICAL APPROVAL

Ethical approval is not applicable to this review article.

## Data Availability

Data sharing does not apply to this article as no new datasets were generated or analysed during the current study.

## References

[1] Bray F , Laversanne M , Sung H , et al. Global cancer statistics 2022: gLOBOCAN estimates of incidence and mortality worldwide for 36 cancers in 185 countries. CA Cancer J Clin. 2024;74:229‐263. 38572751 10.3322/caac.21834

[2] Morgan E , Arnold M , Gini A , et al. Global burden of colorectal cancer in 2020 and 2040: incidence and mortality estimates from GLOBOCAN. Gut. 2023;72:338‐344. 36604116 10.1136/gutjnl-2022-327736

[3] Diaz LA , Shiu K , Kim T , et al. Pembrolizumab versus chemotherapy for microsatellite instability‐high or mismatch repair‐deficient metastatic colorectal cancer (KEYNOTE‐177): final analysis of a randomised, open‐label, phase 3 study. Lancet Oncol. 2022;23:659‐670. 35427471 10.1016/S1470-2045(22)00197-8 PMC9533375

[4] Scott S , Fu J , Chang P . Dopamine receptor D2 confers colonization resistance via microbial metabolites. Nature. 2024;628:180‐185. 38480886 10.1038/s41586-024-07179-5 PMC11097147

[5] Li C , Zhang P , Xie Y , et al. Enterococcus‐derived tyramine hijacks α2A‐adrenergic receptor in intestinal stem cells to exacerbate colitis. Cell Host Microbe. 2024;32:950‐963.e8. 38788722 10.1016/j.chom.2024.04.020

[6] Ji H , Yan X , Zhang L , et al. Prebiotics empower probiotics with gastrointestinal stress resistance for colon‐targeted release to synergistically alleviate colitis. J Control Release. 2025;380:297‐316. 39900225 10.1016/j.jconrel.2025.01.059

[7] Nie Q , Luo X , Wang K , et al. Gut symbionts alleviate MASH through a secondary bile acid biosynthetic pathway. Cell. 2024;187:2717‐2734.e33. 38653239 10.1016/j.cell.2024.03.034

[8] Cui W , Guo M , Liu D , et al. Gut microbial metabolite facilitates colorectal cancer development via ferroptosis inhibition. Nat Cell Biol. 2024;26:124‐137. 38168770 10.1038/s41556-023-01314-6

[9] Zhu Y , Ding W , Chen Y , et al. Genetically encoded bioorthogonal tryptophan decaging in living cells. Nat Chem. 2024;16:533‐542. 38418535 10.1038/s41557-024-01463-7

[10] Xie K , Wang C , Scifo E , et al. Intermittent fasting boosts sexual behavior by limiting the central availability of tryptophan and serotonin. Cell Metab. 2025;37:1189‐1205.e7. 40157367 10.1016/j.cmet.2025.03.001

[11] Arabia A , Muñoz P , Munné‐Bosch S . Fruit‐specific effects of tryptophan and melatonin as active components to extend the functionality of red fruits during post‐harvest processing. Food Chem. 2025;463:141487. 39369602 10.1016/j.foodchem.2024.141487

[12] Tillmann S , Awwad HM , MacPherson CW , et al. The kynurenine pathway is upregulated by methyl‐deficient diet and changes are averted by probiotics. Mol Nutr Food Res. 2021;65:e2100078. 33686786 10.1002/mnfr.202100078

[13] Bender MJ , McPherson AC , Phelps CM , et al. Dietary tryptophan metabolite released by intratumoral *Lactobacillus reute*ri facilitates immune checkpoint inhibitor treatment. Cell. 2023;186:1846‐1862.e26. 37028428 10.1016/j.cell.2023.03.011 PMC10148916

[14] Chen J , Shi G , Yu L , et al. 3‐HKA Promotes Vascular remodeling after stroke by modulating the activation of A1/A2 reactive astrocytes. Adv Sci (Weinh). 2025;12:e2412667. 39854137 10.1002/advs.202412667 PMC11923925

[15] Marszalek‐Grabska M , Walczak K , Gawel K , et al. Kynurenine emerges from the shadows – current knowledge on its fate and function. Pharmacol Ther. 2021;225:107845. 33831481 10.1016/j.pharmthera.2021.107845

[16] Liu Z , Ciudad M , McGaha T . New insights into tryptophan metabolism in cancer. Trends Cancer. 2025;11:629‐641. 40274457 10.1016/j.trecan.2025.03.008

[17] Xue C , Li G , Zheng Q , et al. Tryptophan metabolism in health and disease. Cell Metab. 2023;35:1304‐1326. 37352864 10.1016/j.cmet.2023.06.004

[18] Xie T , Lv T , Zhang T , et al. Interleukin‐6 promotes skeletal muscle catabolism by activating tryptophan–indoleamine 2,3‐dioxygenase 1–kynurenine pathway during intra‐abdominal sepsis. J Cachexia Sarcopenia Muscle. 2023;14:1046‐1059. 36880228 10.1002/jcsm.13193 PMC10067504

[19] Bilir C , Eskiler G , Bilir F . The cytotoxic effects of indoleamine 2, 3‐dioxygenase inhibitors on triple negative breast cancer cells upon tumor necrosis factor α stimulation. J Cancer Res Ther. 2023;19:S74‐S80. 37147986 10.4103/jcrt.jcrt_2365_21

[20] Wu J , Zeng W , Xie H , et al. Microbiota‐induced alteration of kynurenine metabolism in macrophages drives formation of creeping fat in Crohn's disease. Cell Host Microbe. 2024;32:1927‐1943.e9. 39541945 10.1016/j.chom.2024.10.008

[21] Kannen V , Bader M , Sakita J , Uyemura S , Squire J . The dual role of serotonin in colorectal cancer. Trends Endocrinol Metab. 2020;31:611‐625. 32439105 10.1016/j.tem.2020.04.008

[22] Jiang L , Han D , Hao Y , Song Z , Sun Z , Dai Z . Linking serotonin homeostasis to gut function: nutrition, gut microbiota and beyond. Crit Rev Food Sci Nutr. 2024;64:7291‐7310. 36861222 10.1080/10408398.2023.2183935

[23] Alcaino C , Guccio N , Miedzybrodzka EL , et al. Mechanisms of activation and serotonin release from human enterochromaffin cells. Cell Mol Gastroenterol Hepatol. 2025;19:101610. 40812684 10.1016/j.jcmgh.2025.101610 PMC12547937

[24] Li G , Dong S , Liu C , Yang J , Rensen PCN , Wang Y . Serotonin signaling to regulate energy metabolism: a gut microbiota perspective. Life Metab. 2025;4:loae039. 39926388 10.1093/lifemeta/loae039 PMC11803461

[25] Xiong B , Zhu Y , Tian D , et al. Flux redistribution of central carbon metabolism for efficient production of l ‐tryptophan in *Escherichia coli* . Biotechnol Bioeng. 2021;118:1393‐1404. 33399214 10.1002/bit.27665

[26] Wang J , Xu W , Wang R , Cheng R , Tang Z , Zhang M . The outer membrane protein Amuc_1100 of *Akkermansia muciniphila* promotes intestinal 5‐HT biosynthesis and extracellular availability through TLR2 signalling. Food Funct. 2021;12:3597‐3610. 33900345 10.1039/d1fo00115a

[27] Jia D , Kuang Z , Wang L . The role of microbial indole metabolites in tumor. Gut Microbes. 2024;16:2409209. 39353090 10.1080/19490976.2024.2409209 PMC11445886

[28] Tang X , Wu J , Hu Z , et al. Tryptophan metabolism in inter‐organ communication and its potential applications for disease prevention and control. The Innovation Life. 2025;3:100177.

[29] Wu J , Zhang M , Zhang H , et al. PMN‐MDSC: a culprit behind immunosenescence and increased susceptibility to *Clostridioides difficile* infection during aging. Engineering. 2024;42:59‐73.

[30] Gomez de Agüero M , Ganal‐Vonarburg SC , Fuhrer T , et al. The maternal microbiota drives early postnatal innate immune development. Science. 2016;351:1296‐1302. 26989247 10.1126/science.aad2571

[31] Laurans L , Venteclef N , Haddad Y , et al. Genetic deficiency of indoleamine 2,3‐dioxygenase promotes gut microbiota‐mediated metabolic health. Nat Med. 2018;24:1113‐1120. 29942089 10.1038/s41591-018-0060-4

[32] Catena‐Dell'Osso M , Rotella F , Dell'Osso A , Fagiolini A , Marazziti D . Inflammation, serotonin and major depression. Curr Drug Targets. 2013;14:571‐577. 23531160 10.2174/13894501113149990154

[33] Chen L , Bao C , Wu Y , et al. Tryptophan‐kynurenine metabolism: a link between the gut and brain for depression in inflammatory bowel disease. J Neuroinflammation. 2021;18:135. 34127024 10.1186/s12974-021-02175-2 PMC8204445

[34] Dehhaghi M , Kazemi Shariat Panahi H , Guillemin G . Microorganisms, tryptophan metabolism, and kynurenine pathway: a complex interconnected loop influencing human health status. Int J Tryptophan Res. 2019;12:1178646919852996. 31258331 10.1177/1178646919852996 PMC6585246

[35] Holeček M . Serotonin, kynurenine, and indole pathways of tryptophan metabolism in humans in health and disease. Nutrients. 2026;18(3):507. 41683329 10.3390/nu18030507 PMC12899273

[36] Liu Y , Liu Z , Hu Y , et al. Caffeine enhances antitumor T‐cell activity by suppressing kynurenine pathway in colorectal cancer. Nat Commun. 2025;16:5906. 40595642 10.1038/s41467-025-60958-0 PMC12216989

[37] Zhang X , Liu X , Zhou W , et al. Blockade of IDO‐kynurenine‐AhR Axis Ameliorated Colitis‐Associated Colon Cancer via Inhibiting Immune Tolerance. cell mol gastroenterol Hepatol. 2021;12:1179‐1199. 34087454 10.1016/j.jcmgh.2021.05.018 PMC8445903

[38] Liu H , Xiong X , Zhu W , et al. Gut microbial metabolites in cancer immunomodulation. Mol Cancer. 2025;25:8. 41339918 10.1186/s12943-025-02521-5 PMC12781350

[39] Polonio C , McHale K , Sherr D , Rubenstein D , Quintana F . The aryl hydrocarbon receptor: a rehabilitated target for therapeutic immune modulation. Nat Rev Drug Discov. 2025;24:610‐630. 40247142 10.1038/s41573-025-01172-x PMC12875337

[40] Launay J , Delorme R , Pagan C , Callebert J , Leboyer M , Vodovar N . Impact of IDO activation and alterations in the kynurenine pathway on hyperserotonemia, NAD+ production, and AhR activation in autism spectrum disorder. Transl Psychiatry. 2023;13:380. 38071324 10.1038/s41398-023-02687-w PMC10710433

[41] Li Y , Li Q , Yuan R , Wang Y , Guo C , Wang L . Bifidobacterium breve ‐derived indole‐3‐lactic acid ameliorates colitis‐associated tumorigenesis by directing the differentiation of immature colonic macrophages. Theranostics. 2024;14:2719‐2735. 38773969 10.7150/thno.92350 PMC11103503

[42] DiNatale BC , Murray IA , Schroeder JC , et al. Kynurenic acid is a potent endogenous aryl hydrocarbon receptor ligand that synergistically induces interleukin‐6 in the presence of inflammatory signaling. Toxicol Sci. 2010;115:89‐97. 20106948 10.1093/toxsci/kfq024 PMC2855350

[43] Chen Y , Wang Y , Fu Y , Yin Y , Xu K . Modulating AHR function offers exciting therapeutic potential in gut immunity and inflammation. Cell Biosci. 2023;13:85. 37179416 10.1186/s13578-023-01046-y PMC10182712

[44] Thordardottir S , Hangalapura BN , Hutten T , et al. The aryl hydrocarbon receptor antagonist StemRegenin 1 promotes human plasmacytoid and Myeloid dendritic cell development from CD34^+^ hematopoietic progenitor cells. Stem Cells Dev. 2014;23:955‐967. 24325394 10.1089/scd.2013.0521

[45] Contador‐Troca M , Alvarez‐Barrientos A , Barrasa E , et al. The dioxin receptor has tumor suppressor activity in melanoma growth and metastasis. Carcinogenesis. 2013;34:2683‐2693. 23843039 10.1093/carcin/bgt248

[46] Murray I , Patterson A , Perdew G . Aryl hydrocarbon receptor ligands in cancer: friend and foe. Nat Rev Cancer. 2014;14:801‐814. 25568920 10.1038/nrc3846 PMC4401080

[47] Mezrich JD , Fechner JH , Zhang X , Johnson BP , Burlingham WJ , Bradfield CA . An interaction between kynurenine and the aryl hydrocarbon receptor can generate regulatory T cells. J Immunol. 2010;185:3190‐3198. 20720200 10.4049/jimmunol.0903670 PMC2952546

[48] Nguyen NT , Kimura A , Nakahama T , et al. Aryl hydrocarbon receptor negatively regulates dendritic cell immunogenicity via a kynurenine‐dependent mechanism. Proc Natl Acad Sci U S A. 2010;107:19961‐19966. 21041655 10.1073/pnas.1014465107 PMC2993339

[49] Ay EN , Demirkol Ş , Hakan MT , et al. Investigation of possible associations between tryptophan/kynurenine status and FOXP3 expression in colorectal cancer. Scand J Clin Lab Invest. 2022;82:185‐191. 35452343 10.1080/00365513.2022.2040050

[50] Badawy A . Tryptophan metabolism and disposition in cancer biology and immunotherapy. Biosci Rep. 2022;42(11):BSR20221682. 36286592 10.1042/BSR20221682 PMC9653095

[51] Churchhouse AMD , Billard CV , Suzuki T , et al. Loss of DOCK2 potentiates inflammatory bowel disease–associated colorectal cancer via immune dysfunction and IFNγ induction of IDO1 expression. Oncogene. 2024;43:3094‐3107. 39242821 10.1038/s41388-024-03135-9 PMC11473400

[52] Dzhalilova D , Zolotova N , Fokichev N , Makarova O . Murine models of colorectal cancer: the azoxymethane (AOM)/dextran sulfate sodium (DSS) model of colitis‐associated cancer. PeerJ. 2023;11:e16159. 37927787 10.7717/peerj.16159 PMC10624171

[53] Wu X , Wei J , Ran W , et al. The gut microbiota‐xanthurenic acid‐aromatic hydrocarbon receptor axis mediates the anticolitic effects of trilobatin. Adv Sci (Weinh). 2025;12:e2412234. 39836604 10.1002/advs.202412234 PMC11904984

[54] Zhang Y , Yang M , Yang S , et al. Wumei Wan remodels the tumor immune microenvironment via the Ido1‐KYN‐AHR axis in AOM/DSS‐induced colorectal cancer model. J Ethnopharmacol. 2025;353:120371. 40780480 10.1016/j.jep.2025.120371

[55] Niu R , Dong Y , Tong J , et al. NAD^+^ ‐dependent enzyme SIRT3 limits intestinal epithelial cell functions through NAD^+^ synthesis pathway in colorectal cancer. Adv Sci (Weinh). 2026;13:e12532. 41487042 10.1002/advs.202512532 PMC12970194

[56] Brandacher G , Perathoner A , Ladurner R , et al. Prognostic value of indoleamine 2,3‐dioxygenase expression in colorectal cancer: effect on tumor‐infiltrating T cells. Clin Cancer Res. 2006;12:1144‐1151. 16489067 10.1158/1078-0432.CCR-05-1966

[57] Ferdinande L , Decaestecker C , Verset L , et al. Clinicopathological significance of indoleamine 2,3‐dioxygenase 1 expression in colorectal cancer. Br J Cancer. 2012;106:141‐147. 22108515 10.1038/bjc.2011.513 PMC3251860

[58] Lee R , Li J , Li J , et al. Synthetic essentiality of tryptophan 2,3‐dioxygenase 2 in APC ‐mutated colorectal cancer. Cancer Discov. 2022;12:1702‐1717. 35537038 10.1158/2159-8290.CD-21-0680 PMC9262860

[59] Yin J , Yang S , Liu Z , et al. Regulating tumor metabolic reprogramming with biomimetic co‐delivery of simvastatin and kynureninase for immunotherapy. Adv Sci (Weinh). 2026;13:e08107. 41432057 10.1002/advs.202508107 PMC12948188

[60] Hiraku Y , Inoue S , Oikawa S , et al. Metal‐mediated oxidative damage to cellular and isolated DNA by certain tryptophan metabolites. Carcinogenesis. 1995;16:349‐356. 7859368 10.1093/carcin/16.2.349

[61] Bostian A , Eoff R . Aberrant kynurenine signaling modulates DNA replication stress factors and promotes genomic instability in gliomas. Chem Res Toxicol. 2016;29:1369‐1380. 27482758 10.1021/acs.chemrestox.6b00255 PMC5129620

[62] Munn D , Mellor A . Indoleamine 2,3 dioxygenase and metabolic control of immune responses. Trends Immunol. 2013;34:137‐143. 23103127 10.1016/j.it.2012.10.001 PMC3594632

[63] Manlapat A , Kahler D , Chandler P , Munn D , Mellor A . Cell‐autonomous control of interferon type I expression by indoleamine 2,3‐dioxygenase in regulatory CD19^+^ dendritic cells. Eur J Immunol. 2007;37:1064‐1071. 17343295 10.1002/eji.200636690

[64] Badawy A , Guillemin G . The plasma [kynurenine]/[tryptophan] ratio and indoleamine 2,3‐dioxygenase: time for appraisal. Int J Tryptophan Res. 2019;12:1178646919868978. 31488951 10.1177/1178646919868978 PMC6710706

[65] Munn DH , Sharma MD , Hou D , et al. Expression of indoleamine 2,3‐dioxygenase by plasmacytoid dendritic cells in tumor‐draining lymph nodes. J Clin Invest. 2004;114:280‐290. 15254595 10.1172/JCI21583 PMC449750

[66] Ravishankar B , Liu H , Shinde R , et al. Tolerance to apoptotic cells is regulated by indoleamine 2,3‐dioxygenase. Proc Natl Acad Sci U S A. 2012;109:3909‐3914. 22355111 10.1073/pnas.1117736109 PMC3309765

[67] Damerell V , Klaassen‐Dekker N , Brezina S , et al. Circulating tryptophan–kynurenine pathway metabolites are associated with all‐cause mortality among patients with stage I–III colorectal cancer. Int J Cancer. 2025;156:552‐565. 39308420 10.1002/ijc.35183 PMC11621991

[68] Zor DS , Hakan MT , Özgür E , et al. Plasma levels of kynurenine, soluble OX40 and Mir‐138‐5p are associated with tumor‐infiltrating lymphocytes in colorectal cancer: an exploratory study. Anticancer Res. 2023;43:3281‐3288. 37351968 10.21873/anticanres.16503

[69] Li W , Zhou Q , Lv B , et al. *Ganoderma lucidum* polysaccharide supplementation significantly activates T‐cell‐mediated antitumor immunity and enhances anti‐PD‐1 immunotherapy efficacy in colorectal cancer. J Agric Food Chem. 2024;72:12072‐12082. 38750669 10.1021/acs.jafc.3c08385

[70] Sun X , Zhao D , Zhou Y , Wang Q , Qin G , Yao S . Alteration of fecal tryptophan metabolism correlates with shifted microbiota and may be involved in pathogenesis of colorectal cancer. World J Gastroenterol. 2020;26:7173‐7190. 33362375 10.3748/wjg.v26.i45.7173 PMC7723673

[71] Shi D , Wu X , Jian Y , et al. USP14 promotes tryptophan metabolism and immune suppression by stabilizing IDO1 in colorectal cancer. Nat Commun. 2022;13:5644. 36163134 10.1038/s41467-022-33285-x PMC9513055

[72] Wu Z , Wang H , Zheng Z , et al. IDO1 inhibition enhances CLDN18.2‐CAR‐T cell therapy in gastrointestinal cancers by overcoming kynurenine‐mediated metabolic suppression in the tumor microenvironment. J Transl Med. 2025;23:275. 40045363 10.1186/s12967-025-06276-x PMC11884131

[73] Zhang Y‐Y , Han Y , Tan Y‐T , et al. Peritumoural adipose tissue promotes ferroptosis resistance by 3‐hydroxykynurenine‐mediated suppression of ferritinophagy. Nat Cell Biol. 2026;28:783‐796. 41813885 10.1038/s41556-026-01907-x PMC13086588

[74] Guangzhao L , Xin W , Miaoqing W , et al. IDO1 inhibitor enhances the effectiveness of PD‐1 blockade in microsatellite stable colorectal cancer by promoting macrophage pro‐inflammatory phenotype polarization. Cancer Immunol Immunother. 2025;74:71. 39751692 10.1007/s00262-024-03925-w PMC11699167

[75] Liang R , Ding D , Li Y , et al. HDACi combination therapy with IDO1i remodels the tumor microenvironment and boosts antitumor efficacy in colorectal cancer with microsatellite stability. J Nanobiotechnology. 2024;22:753. 39676171 10.1186/s12951-024-02936-0 PMC11648303

[76] Nayak‐Kapoor A , Hao Z , Sadek R , et al. Phase Ia study of the indoleamine 2,3‐dioxygenase 1 (IDO1) inhibitor navoximod (GDC‐0919) in patients with recurrent advanced solid tumors. J Immunother Cancer. 2018;6:61. 29921320 10.1186/s40425-018-0351-9 PMC6009946

[77] Jung KH , LoRusso P , Burris H , et al. Phase I study of the indoleamine 2,3‐dioxygenase 1 (IDO1) inhibitor navoximod (GDC‐0919) administered with PD‐L1 inhibitor (atezolizumab) in advanced solid tumors. Clin Cancer Res. 2019;25:3220‐3228. 30770348 10.1158/1078-0432.CCR-18-2740 PMC7980952

[78] Lorentzen C , Kjeldsen J , Ehrnrooth E , Andersen M , Marie Svane I . Long‐term follow‐up of anti‐PD‐1 naïve patients with metastatic melanoma treated with IDO/PD‐L1 targeting peptide vaccine and nivolumab. J Immunother Cancer. 2023;11:e006755. 37217243 10.1136/jitc-2023-006755 PMC10230976

[79] Lukas RV , Zhai L , Lauing KL , et al. Phase I evaluation of patients with newly diagnosed glioblastoma treated with radiation, nivolumab, and IDO1 enzyme inhibitor BMS‐986205. Clin Cancer Res. 2026;32(16):3476‐3492. 42189896 10.1158/1078-0432.CCR-26-1124 PMC13473868

[80] Sadik A , Somarribas Patterson LF , Öztürk S , et al. IL4I1 is a metabolic immune checkpoint that activates the AHR and promotes tumor progression. Cell. 2020;182:1252‐1270.e34. 32818467 10.1016/j.cell.2020.07.038

[81] Long GV , Dummer R , Hamid O , et al. Epacadostat plus pembrolizumab versus placebo plus pembrolizumab in patients with unresectable or metastatic melanoma (ECHO‐301/KEYNOTE‐252): a phase 3, randomised, double‐blind study. Lancet Oncol. 2019;20:1083‐1097. 31221619 10.1016/S1470-2045(19)30274-8

[82] Fujiwara Y , Kato S , Nesline MK , et al. Indoleamine 2,3‐dioxygenase (IDO) inhibitors and cancer immunotherapy. Cancer Treat Rev. 2022;110:102461. 36058143 10.1016/j.ctrv.2022.102461 PMC12187009

[83] Liu C‐Y , Huang T‐T , Chen J‐L , et al. Significance of kynurenine 3‐monooxygenase expression in colorectal cancer. Front Oncol. 2021;11:620361. 33937026 10.3389/fonc.2021.620361 PMC8085544

[84] Triplett TA , Garrison KC , Marshall N , et al. Reversal of indoleamine 2,3‐dioxygenase–mediated cancer immune suppression by systemic kynurenine depletion with a therapeutic enzyme. Nat Biotechnol. 2018;36:758‐764. 30010674 10.1038/nbt.4180 PMC6078800

[85] Agus A , Planchais J , Sokol H . Gut microbiota regulation of tryptophan metabolism in health and disease. Cell Host Microbe. 2018;23:716‐724. 29902437 10.1016/j.chom.2018.05.003

[86] Sun P , Wang M , Liu Y‐X , et al. High‐fat diet‐disturbed gut microbiota‐colonocyte interactions contribute to dysregulating peripheral tryptophan‐kynurenine metabolism. Microbiome. 2023;11:154. 37468922 10.1186/s40168-023-01606-x PMC10355067

[87] Xue Y , Xiao H , Guo S , et al. Indoleamine 2,3‐dioxygenase expression regulates the survival and proliferation of *Fusobacterium nucleatum* in THP‐1‐derived macrophages. Cell Death Dis. 2018;9:355. 29500439 10.1038/s41419-018-0389-0 PMC5834448

[88] Gao H , Li W , Xu S , et al. Gasdermin D promotes development of intestinal tumors through regulating IL‐1β release and gut microbiota composition. Cell Commun Signal. 2024;22:511. 39434144 10.1186/s12964-024-01890-6 PMC11492562

[89] Vujkovic‐Cvijin I , Dunham RM , Iwai S , et al. Dysbiosis of the gut microbiota is associated with HIV disease progression and tryptophan catabolism. Sci Transl Med. 2013;5:193ra191. 10.1126/scitranslmed.3006438 PMC4094294 23843452

[90] Wu D , Zhu Y . Role of kynurenine in promoting the generation of exhausted CD8(+) T cells in colorectal cancer. Am J Transl Res. 2021;13:1535‐1547. 33841677 PMC8014392

[91] Li F , Xu T , Ling Y , Xu J , Zhang X , Sun S . Combine immune checkpoint indoleamine 2,3‐dioxygenase 1 knockdown with ferroptosis inducer Erastin/RSL3 accelerates colorectal cancer cell ferroptosis. Cytojournal. 2026;23:32. 42291098 10.25259/Cytojournal_262_2024 PMC13260877

[92] Bishnupuri KS , Alvarado DM , Khouri AN , et al. IDO1 and kynurenine pathway metabolites activate PI3K‐Akt signaling in the neoplastic colon epithelium to promote cancer cell proliferation and inhibit apoptosis. Cancer Res. 2019;79:1138‐1150. 30679179 10.1158/0008-5472.CAN-18-0668 PMC6420842

[93] Xie H , Yang J , Wu J , et al. Kynurenine mediates the chemotherapy‐induced intestinal toxicity through modulation of gut microbiota. Nat Commun. 2026;17(1):2087. 41593057 10.1038/s41467-026-68741-5 PMC12953885

[94] Hwang I , Nam JE , Choi W , et al. Serotonin regulates lipogenesis and endoplasmic reticulum stress in alcoholic liver disease. Diabetes Metab J. 2025;49:798‐811. 39905656 10.4093/dmj.2024.0215 PMC12270569

[95] Yoo Y , Joo S . Serotonin influences insulin secretion in rat insulinoma INS‐1E cells. Int J Mol Sci. 2024;25:6828. 38999937 10.3390/ijms25136828 PMC11241493

[96] Singh I , Seth A , Billesbølle CB , et al. Structure‐based discovery of conformationally selective inhibitors of the serotonin transporter. Cell. 2023;186:2160‐2175.e17. 37137306 10.1016/j.cell.2023.04.010 PMC10306110

[97] Toso A , Reinartz S , Pulecchi F , Diamond M . Time coding in rat dorsolateral striatum. Neuron. 2023;111:585‐594. 36796328 10.1016/j.neuron.2023.01.012

[98] Qi P , Chen X , Liu H , Ma J , Qi Z , Xie X . Regulatory mechanisms of gut homeostasis and bone metabolism interplay in osteoporosis. Phenomics. 2025;5:435‐445. 41001429 10.1007/s43657-024-00207-4 PMC12457256

[99] Liu D , Liang C‐H , Huang B , et al. Tryptophan metabolism acts as a new anti‐ferroptotic pathway to mediate tumor growth. Adv Sci (Weinh). 2023;10:e2204006. 36627132 10.1002/advs.202204006 PMC9951368

[100] Gheorghe CE , Leigh S‐J , Tofani GSS , et al. The microbiota drives diurnal rhythms in tryptophan metabolism in the stressed gut. Cell Rep. 2024;43:114079. 38613781 10.1016/j.celrep.2024.114079

[101] Tutton P , Barkla D . The influence of serotonin on the mitotic rate in the colonic crypt epithelium and in colonic adenocarcinoma in rats. Clin Exp Pharmacol Physiol. 1978;5:91‐94. 25153 10.1111/j.1440-1681.1978.tb00657.x

[102] Zhu P , Lu T , Chen Z , et al. 5‐hydroxytryptamine produced by enteric serotonergic neurons initiates colorectal cancer stem cell self‐renewal and tumorigenesis. Neuron. 2022;110:2268‐2282.e4. 35550066 10.1016/j.neuron.2022.04.024

[103] Li T , Fu B , Zhang X , et al. Overproduction of gastrointestinal 5‐HT promotes colitis‐associated colorectal cancer progression via enhancing NLRP3 inflammasome activation. Cancer Immunol Res. 2021;9:1008‐1023. 34285037 10.1158/2326-6066.CIR-20-1043

[104] Li T , Wei L , Zhang X , et al. Serotonin receptor HTR2B facilitates colorectal cancer metastasis via CREB1–ZEB1 axis–mediated epithelial–mesenchymal transition. Mol Cancer Res. 2024;22:538‐554. 38381131 10.1158/1541-7786.MCR-23-0513

[105] Lee J‐Y , Park S , Park EJ , et al. Inhibition of HTR2B‐mediated serotonin signaling in colorectal cancer suppresses tumor growth through ERK signaling. Biomed Pharmacother. 2024;179:117428. 39255737 10.1016/j.biopha.2024.117428

[106] Ling T , Dai Z , Wang H , et al. Serotonylation in tumor‐associated fibroblasts contributes to the tumor‐promoting roles of serotonin in colorectal cancer. Cancer Lett. 2024;600:217150. 39097134 10.1016/j.canlet.2024.217150

[107] Yu H , Qu T , Yang J , Dai Q . Serotonin acts through YAP to promote cell proliferation: mechanism and implication in colorectal cancer progression. Cell Commun Signal. 2023;21:75. 37046308 10.1186/s12964-023-01096-2 PMC10100184

[108] Shimi G . Tryptophan Metabolism in Obesity: pathways, Mechanisms, and Therapeutic Perspectives. Curr Med Sci. 2025;45:1304‐1318. 41201781 10.1007/s11596-025-00136-x

[109] Yano JM , Yu K , Donaldson GP , et al. Indigenous bacteria from the gut microbiota regulate host serotonin biosynthesis. Cell. 2015;161:264‐276. 25860609 10.1016/j.cell.2015.02.047 PMC4393509

[110] Sjögren K , Engdahl C , Henning P , et al. The gut microbiota regulates bone mass in mice. J Bone Miner Res. 2012;27:1357‐1367. 22407806 10.1002/jbmr.1588 PMC3415623

[111] Liu B , Fan L , Wang Y , et al. Gut microbiota regulates host melatonin production through epithelial cell MyD88. Gut Microbes. 2024;16:2313769. 38353638 10.1080/19490976.2024.2313769 PMC10868534

[112] Ahmadi S , Taghizadieh M , Mehdizadehfar E , et al. Gut microbiota in neurological diseases: melatonin plays an important regulatory role. Biomed Pharmacother. 2024;174:116487. 38518598 10.1016/j.biopha.2024.116487

[113] Mao L , Xin F , Ren J , et al. 5‐HT2B‐mediated serotonin activation in enterocytes suppresses colitis‐associated cancer initiation and promotes cancer progression. Theranostics. 2022;12:3928‐3945. 35664068 10.7150/thno.70762 PMC9131283

[114] Sakita JY , Bader M , Santos ES , et al. Serotonin synthesis protects the mouse colonic crypt from DNA damage and colorectal tumorigenesis. J Pathol. 2019;249:102‐113. 31038736 10.1002/path.5285

[115] Wen Y , Tang L , Duan W , et al. Targeting peripheral 5‐HT2AR enhances antitumor immunity in colorectal cancer. Cell. 2026;189(18):5688‐5705.e13. 42551424 10.1016/j.cell.2026.07.028

[116] Wang X , Fu S‐Q , Yuan X , et al. A GAPDH serotonylation system couples CD8+ T cell glycolytic metabolism to antitumor immunity. Mol Cell. 2024;84:760‐775.e7. 38215751 10.1016/j.molcel.2023.12.015

[117] Zhang N , Sundquist J , Sundquist K , Ji J . Use of Selective Serotonin Reuptake Inhibitors Is Associated with a Lower Risk of Colorectal Cancer among People with Family History. Cancers (Basel). 2022;14:5905. 36497383 10.3390/cancers14235905 PMC9741129

[118] Sui H , Xu H , Ji Q , et al. 5‐hydroxytryptamine receptor (5‐HT1DR) promotes colorectal cancer metastasis by regulating Axin1/β‐catenin/MMP‐7 signaling pathway. Oncotarget. 2015;6:25975‐25987. 26214021 10.18632/oncotarget.4543 PMC4694879

[119] Jun J , Oh J , Shim J , Kwak Y , Cho J . Effects of bisphenol A on the proliferation, migration, and tumor growth of colon cancer cells: in vitro and in vivo evaluation with mechanistic insights related to ERK and 5‐HT3. Food Chem Toxicol. 2021;158:112662. 34743013 10.1016/j.fct.2021.112662

[120] Li B , Elsten‐Brown J , Li M , et al. Serotonin transporter inhibits antitumor immunity through regulating the intratumoral serotonin axis. Cell. 2025;188:3823‐3842.e21. 40403728 10.1016/j.cell.2025.04.032 PMC12255530

[121] Ben‐Aharon I , Rotem R , Melzer‐Cohen C , et al. Pharmaceutical agents as potential drivers in the development of early‐onset colorectal cancer: case‐control study. JMIR Public Health Surveill. 2023;9:e50110. 37933755 10.2196/50110 PMC10753427

[122] Sugimura N , Li Q , Chu ESH , et al. *Lactobacillus gallinarum* modulates the gut microbiota and produces anti‐cancer metabolites to protect against colorectal tumourigenesis. Gut. 2021;71:2011‐2021. 34937766 10.1136/gutjnl-2020-323951 PMC9484392

[123] Zhang S , Peng L , Goswami S , et al. Intestinal crypt microbiota modulates intestinal stem cell turnover and tumorigenesis via indole acetic acid. Nat Microbiol. 2025;10:765‐783. 39972061 10.1038/s41564-025-01937-5

[124] Wang J , Hao Y , Yang Y , Zhang Y , Xu C , Yang R , et al. Gut microbiota derived indole‐3‐acetic acid ameliorates precancerous inflammatory intestinal milieu to inhibit tumorigenesis through IL‐35. J Immunother Cancer. 2025;13:e011155. 40274281 10.1136/jitc-2024-011155 PMC12020765

[125] Tomii A , Higa M , Naito K , et al. Activation of the TLR4‐JNK but not the TLR4‐ERK pathway induced by indole‐3‐acetic acid exerts anti‐proliferative effects on Caco‐2 cells. Biosci Biotechnol Biochem. 2023;87:839‐849. 37147026 10.1093/bbb/zbad055

[126] Wang H , Dai S , Xie Y , et al. Colorectal cancer cell's weapon: rNF32 engages SPP1^+^ macrophages to foster liver metastasis, targeted by indole‐3‐acetic acid. Adv Sci (Weinh). 2026;13:e19735. 41387204 10.1002/advs.202519735 PMC12931155

[127] Wang A , Guan C , Wang T , Mu G , Tuo Y . *Lactiplantibacillus plantarum*‐derived indole‐3‐lactic acid ameliorates intestinal barrier integrity through the AhR/Nrf2/NF‐kappaB axis. J Agric Food Chem. 2024;72(16):9236‐9246. 10.1021/acs.jafc.4c01622 38597152

[128] Zhou S , Wang K , Huang J , et al. Indole‐3‐lactic acid suppresses colorectal cancer via metabolic reprogramming. Gut Microbes. 2025;17:2508949. 40409349 10.1080/19490976.2025.2508949 PMC12118437

[129] Han J‐X , Tao Z‐H , Wang J‐L , et al. Microbiota‐derived tryptophan catabolites mediate the chemopreventive effects of statins on colorectal cancer. Nat Microbiol. 2023;8:919‐933. 37069401 10.1038/s41564-023-01363-5

[130] Wang L , Tu Y‐X , Chen L , et al. Black rice diet alleviates colorectal cancer development through modulating tryptophan metabolism and activating AHR pathway. Imeta. 2024;3:e165. 38868519 10.1002/imt2.165 PMC10989083

[131] Zhang Q , Zhao Q , Li T , et al. Lactobacillus plantarum‐derived indole‐3‐lactic acid ameliorates colorectal tumorigenesis via epigenetic regulation of CD8+ T cell immunity. Cell Metab. 2023;35:943‐960.e9. 37192617 10.1016/j.cmet.2023.04.015

[132] Zhou T , Wu J , Khan A , et al. A probiotic *Limosilactobacillus fermentum* GR‐3 mitigates colitis‐associated tumorigenesis in mice via modulating gut microbiome. NPJ Sci Food. 2024;8:61. 39242568 10.1038/s41538-024-00307-5 PMC11379937

[133] Xu H , Wang Y , Liu G , et al. Nano‐armed *Limosilactobacillus reuteri* for enhanced photo‐immunotherapy and microbiota tryptophan metabolism against colorectal cancer. Adv Sci (Weinh). 2025;12:e2410011. 39739630 10.1002/advs.202410011 PMC11831460

[134] Jia D , Wang Q , Qi Y , et al. Microbial metabolite enhances immunotherapy efficacy by modulating T cell stemness in pan‐cancer. Cell. 2024;187:1651‐1665.e21. 38490195 10.1016/j.cell.2024.02.022

[135] Ishii K , Naito K , Tanaka D , Koto Y , Kurata K , Shimizu H . Molecular mechanisms of skatole‐induced inflammatory responses in intestinal epithelial caco‐2 cells: implications for colorectal cancer and inflammatory bowel disease. Cells. 2024;13:1730. 39451248 10.3390/cells13201730 PMC11505633

[136] Kurata K , Ishii K , Koto Y , Naito K , Yuasa K , Shimizu H . Skatole‐induced p38 and JNK activation coordinately upregulates, whereas AhR activation partially attenuates TNFα expression in intestinal epithelial cells. Biosci Biotechnol Biochem. 2023;87:611‐619. 36941128 10.1093/bbb/zbad030

[137] Śmiełowska M , Ligor T , Kupczyk W , Szeliga J , Jackowski M , Buszewski B . Screening for volatile biomarkers of colorectal cancer by analyzing breath and fecal samples using thermal desorption combined with GC‐MS (TD‐GC‐MS). J Breath Res. 2023;17:047102. 10.1088/1752-7163/ace46e 37406626

[138] Ichisaka Y , Takei C , Naito K , et al. The role of indoxyl sulfate in exacerbating colorectal cancer during chronic kidney disease progression: insights into the Akt/β‐catenin/c‐Myc and AhR/c‐Myc pathways in HCT‐116 colorectal cancer cells. Toxins (Basel). 2025;17:17. 39852970 10.3390/toxins17010017 PMC11769072

[139] Song Qi , Jin Z , Zhang H , et al. Fusobacterium nucleatum‐derived 3‐indolepropionic acid promotes colorectal cancer progression via aryl hydrocarbon receptor activation in macrophages. Chem Biol Interact. 2025;414:111495. 40174685 10.1016/j.cbi.2025.111495

[140] Mohammadzadeh R , Mahnert A , Zurabishvili T , et al. Cross‐domain metabolic interactions link *Methanobrevibacter smithii* to colorectal cancer microbial ecosystems. Nat Commun. 2026;17(1):2979. 41720792 10.1038/s41467-026-69711-7 PMC13035836

[141] Venkateswaran N , Garcia R , Lafita‐Navarro MC , et al. Tryptophan fuels MYC‐dependent liver tumorigenesis through indole 3‐pyruvate synthesis. Nat Commun. 2024;15:4266. 38769298 10.1038/s41467-024-47868-3 PMC11106337

[142] Dai Z , Deng K‐L , Wang X‐M , Yang D‐X , Tang C‐L , Zhou Y‐P . Bidirectional effects of the tryptophan metabolite indole‐3‐acetaldehyde on colorectal cancer. World J Gastrointest Oncol. 2024;16:2697‐2715. 38994159 10.4251/wjgo.v16.i6.2697 PMC11236226

[143] Sun H , Zhang A‐H , Zhang H‐L , et al. Ultra‐performance liquid chromatography/mass spectrometry technology and high‐throughput metabolomics for deciphering the preventive mechanism of mirabilite on colorectal cancer via the modulation of complex metabolic networks. RSC Adv. 2019;9:35356‐35363. 35528071 10.1039/c9ra07687e PMC9074663

[144] Fong W , Li Q , Ji F , et al. Lactobacillus gallinarum ‐derived metabolites boost anti‐PD1 efficacy in colorectal cancer by inhibiting regulatory T cells through modulating IDO1/Kyn/AHR axis. Gut. 2023;72:2272‐2285. 37770127 10.1136/gutjnl-2023-329543 PMC10715476

